# Optimization and comparison of different methods for assessing cell viability in intervertebral disc organ cultures

**DOI:** 10.3389/fbioe.2026.1796998

**Published:** 2026-04-29

**Authors:** Marcia Muerner, Junxuan Ma, Barbora Kubincova, Christoph M. Sprecher, Martin J. Stoddart, Sibylle Grad

**Affiliations:** 1 AO Research Institute Davos, Davos, Switzerland; 2 ETH Zurich: Eidgenössische Technische Hochschule Zurich, Zurich, Switzerland; 3 Department of Orthopedic Surgery and Traumatology, H-OCH Health Ostschweiz, Cantonal Hospital of St. Gallen, St. Gallen, Switzerland

**Keywords:** calcein AM/EthD-1, cell viability, intervertebral disc, LDH assay, MTT assay, tissue viability

## Abstract

**Introduction:**

Whole-organ intervertebral disc (IVD) culture is widely used to study IVD degeneration and evaluate regenerative therapies. Spatial visualization of resident cell viability is essential, yet commonly used assays, such as Calcein AM/Ethidium Homodimer-1 (EthD-1), lactate dehydrogenase (LDH)/EthD-1, and MTT/DAPI, lack standardized protocols that facilitate implementation.

**Methods:**

In this study, we optimized and compared protocols for all three staining methods in bovine caudal IVDs and evaluated their strengths and limitations using appropriate positive and negative controls.

**Results:**

Selection of suitable controls was critical for reliable viability assessment. Calcein AM/EthD-1 provided a straightforward approach but required protocol modifications, including Collagenase P pre-treatment, to ensure adequate tissue penetration. This method also requires immediate processing and imaging after harvesting. In contrast, LDH/EthD-1 and MTT/DAPI allows for less time-sensitive workflows and enables repeated staining. However, LDH/EthD-1 is unsuitable for short-term experiments, as LDH signal persists for up to 36 h after cell death, except under extreme conditions such as repeated snap-freezing. MTT/DAPI proved more suitable for short-term applications.

**Discussion:**

These findings provide practical guidance for selecting and implementing viability assays in IVD culture, facilitating method choice based on specific experimental objectives and time constraints. Moreover, the insights gained may also be applicable to other dense tissues, such as tendon and cartilage.

## 1 Introduction

Intervertebral disc (IVD) degeneration (IVDD) lacks effective regenerative therapies (Schol et al., 2024). This drives active research efforts aimed at understanding underlying mechanisms and evaluating novel treatments. Whole-organ culture is a widely used and well-established model to study IVD physiology, degeneration, and potential therapies (Salzer et al., 2023; Pfannkuche et al., 2020; Alini et al., 2023; Alini et al., 2008). While fresh whole human discs are scarce, bovine coccygeal IVDs are readily available from abattoirs, where they are by-products of the normal slaughtering process. They offer the advantage of being relatively similar to human discs in size (14–22 mm diameter, 5–10 mm height) and intradiscal pressure (0.1–0.3 MPa) (Alini et al., 2008). Nonetheless, bovine IVDs have certain limitations: They are more cellular than human discs, and slaughtered animals are typically young and healthy. This limits direct translational relevance and often requires experimental induction of degeneration (Pfannkuche et al., 2020). Cell viability, defined as the proportion of healthy cells in a tissue, is a key outcome measure in such studies (Stoddart, 2011). A healthy cell is intact, metabolically active, and able to proliferate. Despite the expanding focus on defined cell death pathways (e.g., apoptosis, necroptosis, ferroptosis), fundamental measures of total cell viability remain vital for interpreting experimental outcomes (Hu et al., 2021). Common single-cell viability assays use a dual staining approach, whereby one stain marks viable cells, while the other marks dead cells. The exact definition of “viable” and “dead” depends on the dye’s specificity (e.g., indicating enzyme activity or membrane integrity). Whereas many commercially available viability assays are well standardized for monolayer cell cultures, working with whole tissue samples such as IVD can pose additional challenges, including limited stain penetration and increased background due to dense tissue (reduced mass transfer and autofluorescence) (Gantenbein-Ritter et al., 2011). In dense tissue, metabolic dyes can also have the complication that the outer edges of the tissue can metabolize the reagent before it is able to diffuse into the center of the explant, leading to false results. Longer staining durations to improve tissue penetration trade off with greater exposure to the often-toxic staining reagents (Stoddart et al., 2006). A specific challenge in evaluating cell viability in the IVD relates to its distinct regional extracellular matrix (ECM) composition: the collagen type I-rich outer annulus fibrosus (oAF), the transitional inner annulus fibrosus (iAF), and the proteoglycan-rich nucleus pulposus (NP). Variations in tissue composition can differentially impact stain diffusion. Additionally, differences in cell morphology and density further hinder consistent and automated viability assessments (fewer and round cells in NP vs. many elongated cells in the oAF). As a result, extensive and time-consuming optimization is often required to achieve reliable and reproducible results.

A widely used approach is the Calcein AM/Ethidium Homodimer-1 (EthD-1) dual staining method. Calcein AM enters viable cells and is converted by esterases into green-fluorescent Calcein, while EthD-1 only enters membrane-compromised cells, binding nucleic acids and fluorescing red (Gantenbein-Ritter et al., 2011). Despite its popularity, the method faces practical challenges due to limited stain penetration and the need for immediate imaging on the day of tissue harvesting. Using small tissue fragments and longer incubation improves penetration but limits assessment to small fragments of the overall tissue, making it hard to detect regional differences comprehensively (Gantenbein-Ritter et al., 2011; Peroglio et al., 2017). Additionally, cutting the tissue into fragments may result in an increased number of falsely classified dead cells due to cutting-induced damage.

An alternative method is based on lactate dehydrogenase (LDH) activity in viable cells. This technique is performed in unfixed cryosections (Vernengo et al., 2023; Zhou et al., 2024; Li et al., 2016). LDH is an enzyme that converts lactate to pyruvate with a tetrazolium salt, producing a blue-purple colored precipitate in (pseudo-)viable cells visible by brightfield microscopy. Again, EthD-1 can be used as counterstain to mark non-viable cells. Advantages include quick initial sample processing, easy dye permeation, flexible timing for sectioning and imaging, use of remaining sections for other analyses (e.g., structural staining), and assessment of large areas, allowing regional differences in one plane to be detected. A limitation is that LDH activity can persist up to 36 h after cell death, potentially overestimating viability (Wong et al., 1987; Stoddart, 2011). Therefore, reliable negative controls are essential.

A third option is MTT (3-(4,5-dimethylthiazol-2-yl)-2,5-diphenyl tetrazolium bromide) staining, also applicable to cryosections. Unlike LDH/EthD-1, MTT must be applied to the tissue before sectioning, typically with 3-h incubation (McDonnell and Buckley, 2021; McDonnell et al., 2023). MTT, a yellow, water-soluble compound, is reduced by mitochondrial enzymes in viable cells to insoluble purple formazan. Accumulated formazan then reflects metabolic activity. DAPI can be used to counterstain all nuclei, fluorescing blue, allowing estimation of viability by comparing MTT-positive cells to total DAPI-stained nuclei (McDonnell and Buckley, 2021).

Although the abovementioned staining techniques are routinely used in IVD tissue analysis, protocol details have not been standardized. This hampers straightforward reproduction and limits comparability among studies. Also, whereas most studies include positive controls, negative controls are often completely omitted, posing a substantial risk of signal misinterpretation due to suboptimal imaging settings.

To improve reliability of future cell viability assessments in IVD research, this work aimed to 1) critically compare protocols for three core viability assessment strategies in assessing IVD viability and to 2) set up suitable positive and negative controls for each technique. Based on our findings, a practical and rational framework is provided, including guidance for assay selection and practical tips to facilitate straightforward implementation in future studies.

## 2 Materials and methods

### 2.1 IVD dissection and processing

Whole IVD specimens were isolated from tails of cattle slaughtered for meat production as previously outlined (for detailed donor information see Supplementary Figure S1) (Vernengo et al., 2023). Briefly, tails were washed using Lifo-Scrub® (B.Braun, Germany) and then immersed in 1% betadine solution (Mundipharma, Germany) for 10 min. Carefully, muscles, tendons, nerves as well as bony processes were removed. Discs were isolated by separating them from the vertebrae at the level of the growth plates. This was achieved either by manual cutting with a scalpel or by using an Exakt 300 band saw (Exakt, Germany). After isolation the discs were cleaned using the Pulsavac wound debridement system (Zimmer Biomet, United States) to remove blood clots. Then, the discs were washed in 10% Pen-Strep (Thermo Fisher, United States) for 12 min and 1% Pen-Strep solution for 2 min. The discs were randomly assigned to the experimental groups, having one disc per tail and group. Disc diameters were measured with calipers four times per disc (twice on both sides) and then averaged, while height was calculated as the average of two measurements. At least three independent donors were included in each experimental group.

The experimental groups, two positive control (viable) groups and four negative control (dead) groups, are summarized in Table 1. Whereas discs from the Freshly harvested group were harvested directly after dissection and cleaning, discs from the Overnight free swelling group were kept overnight in culture medium (DMEM HG with 4.5 g/L glucose supplemented with sodium bicarbonate, pyruvate, 1% Pen-Strep, 2% fetal bovine serum, 1% ITS+, 1% nonessential amino acids, 25 mmol/L HEPES (Gibco, Life Technologies, United States), 50 μg/mL ascorbate-2-phosphate, and 50 μg/mL primocin (Invitrogen, United States)) at 37 °C and 5% CO2 and processed the next day. Negative control discs were subjected to freeze–thaw cycles derived from regimes previously reported for LDH/EthD-1 staining in bone sections (Stoddart, 2011). Discs from the Negative Control 1 group underwent two freeze–thaw cycles (−20 °C ⇔ RT) over 24 h and were harvested after complete thawing. During these cycles, discs were not immersed in liquid. Stoddart et al. suggested that snap-freezing and/or heating for 18 h is sufficient to induce detectable cell death in bone (Stoddart, 2011). To assess whether LDH activity in IVD tissue could also disappear in less than the commonly assumed 36 h after cell death, discs from the Negative Control 2–3 groups were snap-frozen twice in liquid nitrogen and thawed at 37 °C between cycles (∼1 h). Discs were not immersed in liquid during freezing or thawing. Subsequently, they were immersed in 15 mL PBS and heat-incubated at 56 °C for 24, 48, or 72 h (Negative controls 2–4).

**TABLE 1:** Disc conditions tested.

Group	Treatment	Protocols tested	Sample number
Freshly harvested	Harvesting right after dissection and cleaning	Calcein AM/EthD-1, ColP-Calcein AM/EthD-1, MTT/DAPI, LDH/EthD-1	5
Overnight free swelling	Harvesting after 1 day of free swelling in medium	Calcein AM/EthD-1, ColP-Calcein AM/EthD-1, MTT/DAPI, LDH/EthD-1	≥3
Negative control 1	2 freeze-thaw cycles (−20 °C ⇔ RT) over 24 h and harvesting after complete defrosting	Calcein AM/EthD-1, ColP-Calcein AM/EthD-1, MTT/DAPI LDH/EthD-1	3
Negative control 2	2 quick snap-freeze thaw cycles (−196° C ⇔ 37 °C until complete defrosting), followed by a 24-h heat incubation (56 °C) in PBS	ColP-Calcein AM/EthD-1, LDH/EthD-1	3
Negative control 3	2 quick snap-freeze thaw cycles (−196° C ⇔ 37 °C until complete defrosting), followed by a 48-h heat incubation (56 °C) in PBS	ColP-Calcein AM/EthD-1, LDH/EthD-1	3
Negative control 4	2 quick snap-freeze thaw cycles (−196° C ⇔ 37 °C until complete defrosting), followed by a 72-h heat incubation (56 °C) in PBS	ColP-Calcein AM/EthD-1, LDH/EthD-1	3

**FIGURE 1:**
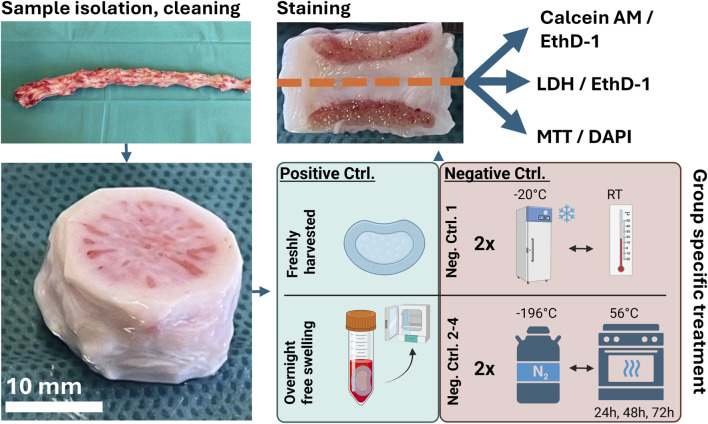
Experimental set-up: Harvesting procedure including disc isolation, cleaning and group specific treatment, followed by a sagittal and transversal cut and respective staining.

Immediately after respective treatment, discs were cut sagitally with a Padget tool and separated further into quarters by a transversal cut of the sagittal halves (Figure 1). Quarters were immediately further processed for the specific viability assessment avoiding time delays or tissue drying.

### 2.2 Calcein AM/EthD-1 staining

The remaining bone of the IVD quarter was removed with a scalpel and tissue was separated into NP, iAF and oAF containing 3-5 x 3-5 x 3–5 mm tissue pieces. Two different protocols for Calcein AM/EthD-1 staining were applied: a protocol based on previous work for IVD tissue staining (Calcein AM/EthD-1) and an adapted version including Collagenase P treatment (ColP-Calcein AM/EthD-1) (Gantenbein-Ritter et al., 2011). Briefly, tissue pieces were immersed in 1 mL staining solution [6.3 µM Calcein AM (Sigma- Aldrich, United States) + 0.8 µM EthD-1 (Sigma- Aldrich, United States)] in serum free medium (DMEM HG with 4.5 g/L glucose supplemented with sodium bicarbonate and pyruvate) for 1 h at 37 °C protected from light. Importantly, Gantenbein et al. used 2-h incubation for Calcein AM/EthD-1 staining (Gantenbein-Ritter et al., 2011). We tested 2-h incubation on one sample and qualitatively observed no difference compared to 1-h incubation (see Supplementary Figure S2). To minimize overall staining time, we therefore opted for 1-h incubation. For samples assigned to the ColP-Calcein AM/EthD-1 group, tissue pieces were immersed in 1 mg/mL Collagenase P (ColP; Roche, Switzerland) diluted in serum free DMEM HG for 1 h at 37 °C before tissue was immersed in the staining solution for 1 h as described above. After staining, tissues were washed twice in PBS and imaged immediately using a LSM800 confocal microscope (Zeiss, Germany) at a 5× objective using two fluorescent channels (EthD-1: 350 nm excitation, 617 nm emission, 1.5% laser power, 38 µm pinhole; Calcein: 494 nm excitation and 514 nm emission, 0.15% laser power, 38 µm pinhole). Detector gain and scanning parameters were kept constant across samples. For imaging, tissue pieces were placed in a custom-made aluminum plate with a circular hole (diameter: 25 mm), as described in (Gantenbein-Ritter et al., 2011), immersed completely in PBS and coverslipped as described previously (Gantenbein-Ritter et al., 2011). Both Calcein AM and EthD-1 signals exhibited minimal background fluorescence, with individual cells clearly distinguishable, indicating that tissue autofluorescence was low. Therefore, no correction strategies for autofluorescence were applied. At least 3 images were taken from each stained tissue piece. Cells stained green (Calcein AM) were regarded as viable, while cells stained red (EthD-1) were considered dead.

### 2.3 LDH/EthD-1 staining

For LDH/EthD-1 staining disc quarters were immersed in optimal cutting temperature compound (OCT; Tissue-Tek, Switzerland) and snap-frozen in a container placed in 2-methylbutane cooled with liquid nitrogen. A cryostat was used to cut 10-micron slides. After PBS washing, all sections were incubated in 1 μg/mL EthD-1 (Sigma-Aldrich, United States) for 45 min in a dark, humid chamber at room temperature. After PBS washes, LDH solution (Polypep, 60 mM Lactic acid, 2.64 mM β-nicotinamide adenine and 3.67 mM nitroblue powder, pH = 8) was added to the sections and incubated for 3 h at 37 °C in a dark humid chamber. Then the sections were washed, fixed in 4% formalin and coverslipped using faramount aqueous mounting medium (Dako, Denmark). Sections were stored horizontally at 4 °C until imaging. Imaging was done on the next day using a BX63 Olympus microscope (Japan) and a 10 X optics. Brightfield was used to visualize LDH (210 µs exposure, 0.5x gain), while red fluorescence (527 nm excitation, 617 nm emission, 200 ms exposure, 0.5x gain) was used to image EthD-1. To assess stain behavior over time, sections of the Freshly harvested and Negative control 2 group were imaged again 10 and 30 days after staining and first imaging. Cells stained positively for LDH (blue in brightfield channel) were considered as viable, whereas cells showing EthD-1 staining were considered dead. In line with previous work, cells displaying both stains were classified as viable (Secerovic et al., 2022; Zhou et al., 2024). This is based on the potential membrane damage of LDH positive cells introduced by the tissue sectioning procedure, leading to dual stain positivity. Nevertheless, there could be a risk that this method may overestimate viability, as cells with compromised membranes positive for EthD-1 that retain an LDH signal are still counted as viable.

### 2.4 MTT/DAPI staining

For MTT/DAPI staining, the remaining endplate of a disc quarter was removed, and the disc tissue was incubated for 3 h at 37 °C on an orbital shaker in 0.5 mg/mL MTT in phenol-free DMEM HG (Gibco, United States). Then tissue was rinsed with PBS and OCT embedded overnight. Afterwards, the tissue was snap-frozen and stored at – 70 °C. Thin sections (10 micron) were cut, and PBS was used to remove OCT. Then sections were fixed in 4% formalin, washed, coverslipped using ProLong™ Gold Antifade mountant with DNA stain DAPI (Invitrogen, United States) and sealed with nail polish. Stained sections were stored horizontally at 4 °C. They were imaged the next day using a BX63 Olympus microscope at a 10X optics. Brightfield was used to image MTT (210 µs exposure, 0.5x gain), whereas a fluorescent channel was used for DAPI detection (345 nm excitation, 455 nm emission, 2.37–5 s exposure, 4x gain). Cells being positive for both MTT and DAPI were considered as viable cells, whereas dead cells were identified when DAPI stain was present without formazan deposition.

### 2.5 Image processing

The resulting images were processed and analyzed using cellSens (Olympus specific software) or Zen Blue (Zeiss specific software), and cells counted in ImageJ. Representative images shown in this manuscript were only modified by cropping and uniform brightness adjustments applied to the entire image. No modifications were made to individual channels or specific regions.

### 2.6 Cell viability counting

To avoid any methodological bias (overestimation/underestimation of cell viability between methods due to different thresholding in automatic counting procedures), all images were counted manually using the ImageJ cell counter plug-in and a total area of 1 mm^2^ (LDH/EthD-1, MTT/DAPI) and 1.44 mm^2^ (Calcein AM/EthD-1) was counted for each image. Cell viability was calculated as percentage of viable cells (Calcein AM, LDH, or MTT positive cells) of total cell number.

### 2.7 Data presentation and statistical analysis

Data are presented as mean ± SD with individual data points annotated. A mixed-effects model with Geisser-Greenhouse correction was fit using Restricted Maximum Likelihood (REML). Fixed effects included IVD region (NP, iAF, oAF), treatment, and the region × treatment interaction. Subject/sample was included as a random effect. Tukey’s multiple comparison test was used to determine group differences. An inter-observer reliability analysis of manual cell viability counts was performed on 47 randomly selected images representing all three staining methods. Cell viability in these images was independently assessed by two researchers. Reliability was evaluated using the single-measure, two-way random-effects intraclass correlation coefficient (ICC2), a Bland–Altman plot, and the mean difference between observers. All analyses were conducted using Python (version 3.12.12) and GraphPad Prism (version 10.1.2).

## 3 Results

### 3.1 Calcein AM/EthD-1

**FIGURE 2:**
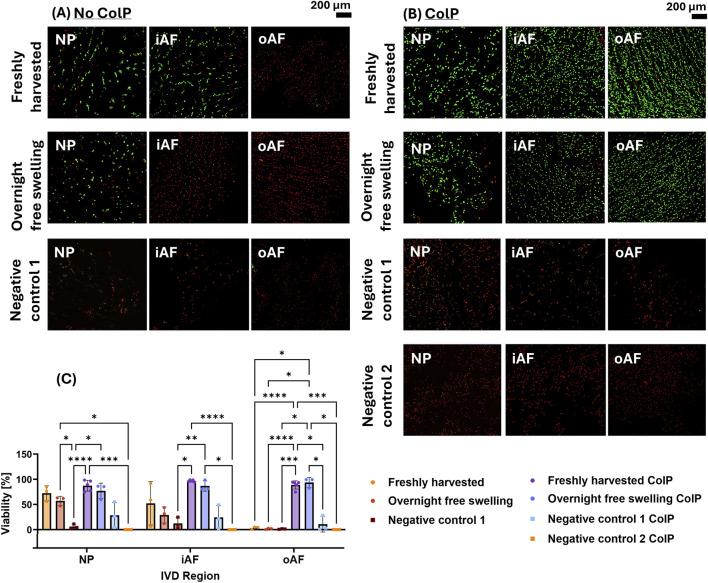
Calcein AM/EthD-1 stained IVD tissue with and without ColP pre-treatment. **(A)** Representative images of Calcein AM/EthD-1 stained IVD tissue without ColP pre-treatment. **(B)** Representative images of ColP-Calcein AM/EthD-1 stained IVD tissue with ColP pre-treatment. **(C)** Quantification of cell viability for the different treatment groups tested. At least three biological replicates were counted per group (N = 3–5). Statistical differences were assessed using a mixed-effect model with Tukey’s multiple comparison test (*p < 0.05, **p < 0.01 ***p < 0.001, ****p < 0.0001).

Cell viability was assessed using both Calcein AM/EthD-1 and ColP-Calcein AM/EthD-1 protocols for Freshly harvested, Overnight free swelling and Negative control 1 discs, while Negative control 2 discs were evaluated only with the ColP-Calcein AM/EthD-1 protocol. Representative images and quantitative results are shown in Figures 2A–C.

The number of Calcein stained cells was low using the traditional staining method, especially for AF tissue, even for Freshly harvested and Overnight free swelling groups. Tissue pre-treatment with ColP led to an increase in efficiency of Calcein staining in the Freshly harvested group and Overnight free swelling group for all IVD regions. This increase was most pronounced in the oAF, where apparent percentage of Calcein stained cells increased from a mean of 3.25%–88.75% and 1.57%–93.57% for the Freshly harvested and Overnight free-swelling groups, when treated with ColP. Since ColP-treatment efficiently enhanced Calcein staining efficiency, we decided to use the ColP-treated method for the following viability evaluations.

Positive Controls: Although the ColP-treated Overnight free-swelling group exhibited approximately 10% lower viability in both iAF and NP tissues compared to the Freshly harvested ColP-treated group, these differences were not statistically significant. Both groups showed viabilities >85% for the ColP–Calcein AM/EthD-1 procedure.

Negative Controls: For ColP-Calcein AM/EthD-1-stained tissue, both tested negative controls (Negative control 1 and 2) showed a drop in cell viability compared to the Freshly harvested tissue (Figure 2C). However, only Negative control 2 samples were found to be significantly less viable compared to Freshly harvested and Overnight free swelling tissue for NP and iAF. Negative control 1 showed a high variability between the individual samples, especially for NP (+/- 25.50%) and iAF (+/- 23.60%), whereas Negative control 2 led to complete cell death in all regions.

### 3.2 MTT/DAPI

**FIGURE 3:**
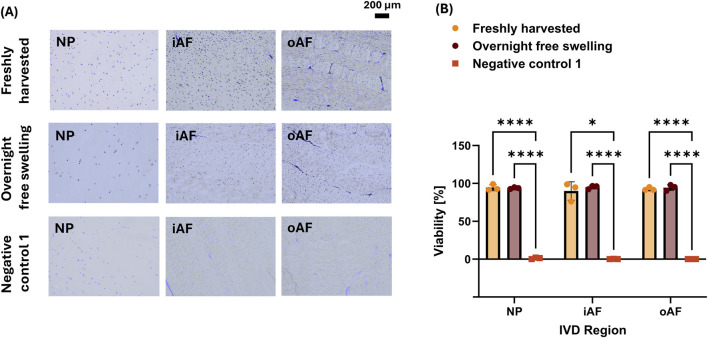
MTT/DAPI stained IVD tissue for Freshly harvested, Overnight free swelling and Negative control 1 IVDs. **(A)** Representative images of all regions. **(B)** Quantification of viability of MTT/DAPI stained tissue. Statistical differences were assessed using a mixed-effect model with Tukey’s multiple comparison test (*p < 0.05, ****p < 0.0001).

MTT/DAPI showed a viability of >92% for Freshly harvested tissue and Overnight free swelling for all regions and a cell viability of <2% for Negative control 1 (Figure 3). In all regions, Negative control 1 was significantly lower than the Freshly harvested and Overnight free-swelling groups.

DAPI fluorescence stability over time was assessed qualitatively by comparing images taken on day 1, 10, and 30 (Supplementary Figure S3). Fluorescence remained strong and no clear decline was observed qualitatively for all IVD regions.

### 3.3 LDH/EthD-1

**FIGURE 4:**
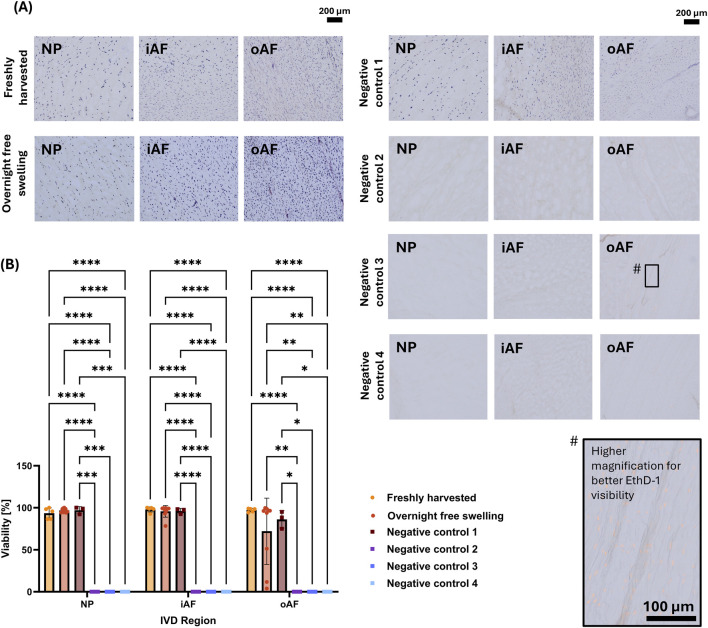
LDH/EthD-1-stained tissue for Freshly harvested, Overnight free swelling, and Negative control 1–4. **(A)** Representative images for all groups and regions analyzed. For better visualization of EthD-1 a magnified example is shown (indicated with #). **(B)** Quantification of all tested groups. At least three biological replicates were counted per group (N = 3–9). Statistical differences were assessed using a mixed-effect model with Tukey’s multiple comparison test (*p < 0.05, **p < 0.01, ***p < 0.001, ****p < 0.0001).

Concerning positive controls, both Freshly harvested and Overnight free swelling discs showed a high viability of >93%, except for the oAF of the Overnight free swelling group (mean viability = 72.04%; Figure 4B). Due to high variation in LDH staining in the oAF of the free swelling group, additional samples were included, and sections from all samples were stained simultaneously (n = 9). Representative images illustrating the observed discrepancies are shown in Supplementary Figure S4. In brief, three out of nine samples showed increased cell death in the oAF, while the remaining six samples appeared viable. Interestingly, the same samples that showed low oAF viability with the LDH method appeared viable when tested using ColP-Calcein AM/EthD-1. Despite these inconsistencies, there were no statistically significant differences between Freshly harvested and Overnight free swelling samples in any region.

To find a suitable negative control, LDH/EthD-1 staining was performed for all negative control groups (Negative control 1–4). Notably, Negative control 1 group appeared viable for all regions (mean viabilities >86%). There was no significant difference to the Freshly harvested or Overnight free swelling group. Negative controls 2-4 resulted in complete loss of LDH stain for all regions.

Potential fading of fluorescent EthD-1 staining over time was qualitatively assessed (Supplementary Figure S5). Imaging at 10- and 30-day post-staining for both the Freshly harvested and Negative control 2 groups demonstrated that samples can be imaged for up to 30 days with appropriate adjustment of exposure. This observation was consistent across all IVD regions.

### 3.4 Inter-observer reliability

Intraclass correlation coefficient was 0.956 with a 95% confidence interval of 0.92–0.98. Mean difference between observers was 0.78% (for Bland-Altman plot see Supplementary Figure S12).

## 4 Discussion

### 4.1 Detailed protocols for optimal reproducibility

Calcein AM/EthD-1 remains widely used to assess cell viability in cell and tissue cultures. For tissues, poor stain penetration in dense 3D matrices is a known challenge. This not only affects viability stains but all types of staining. Moreover, metabolically active substrates used in stainings (e.g., Calcein AM) may be depleted by cells near the surface, making uniform signal distribution throughout the sample difficult to achieve. Stain penetration has previously been shown to improve with extended incubation times and through the implementation of a temperature-cycling protocol. By initially incubating at 4 °C followed by gradual warming, deeper diffusion can be facilitated by diffusion of the stain toward the tissue core before it is depleted at the periphery (Stoddart, 2011). In our studies with bovine tissue, traditional Calcein AM/EthD-1 staining yielded good results for the less dense NP, but unsatisfactory results for the denser oAF. There, Calcein AM failed to penetrate the dense collagen network, leading to strong false negative results (viable cells not stained with Calcein). Interestingly, for the traditional staining method, Calcein staining was low, while EthD-1 staining remained. Since the oAF represents the densest region of the disc, it likely posed the greatest barrier to diffusion. Because the molecular weights of the two molecules are relatively similar (Calcein AM: 994 Da; EthD-1: 856 Da), we hypothesize that the observed differences in diffusion were primarily driven by differences in hydrophilicity and tissue penetration. EthD-1 is water-soluble due to its bipolar nature, whereas Calcein AM, in its esterified form, is known to be lipophilic (Tenopoulou et al., 2007). In addition, IVD tissue carries a net negative charge due to its high proteoglycan content. This may have further facilitated EthD-1 diffusion into the tissue, as EthD-1 carries a strong positive charge. If the assay is to be further optimized, additional studies are required to fully elucidate the mechanisms underlying these differences in diffusion. In our study, adding the enzyme ColP as a pretreatment significantly improved staining quality. By effectively loosening the dense ECM, ColP likely allowed for easier diffusion and better stain penetration. An alternative explanation for differential stain intensity is the variation in metabolic and esterase activity between AF and NP cells. AF cells have been shown to rely more on aerobic metabolism than NP cells (Wang et al., 2021). This elevated aerobic activity may have accelerated stain metabolism, resulting in a pronounced false-negative signal due to early stain depletion. However, after ColP treatment, AF cells were uniformly labelled, suggesting that stain concentration was sufficient for all cells, assuming ColP did not significantly affect the metabolic activity of AF cells. One potential limitation of ColP usage is the fact that enzymes tend to digest selectively dead cells, which could lead to an overestimation of cell viability (Gantenbein-Ritter et al., 2008). However, when comparing Negative control 1 samples with and without ColP treatment, no significant reduction in EthD-1 staining was observed in the ColP-treated group. This suggests that any potential effect of enzymatic degradation on dead cell detection is likely minimal. Another limitation of ColP use is its potential effect on tissue architecture. If tissue is intended for downstream analyses such as histology, ColP treatment may introduce artifacts, particularly in the oAF. Nevertheless, we recommend its use to improve staining quality in certain dense tissues where penetration issues occur.

To avoid penetration issues, staining can be applied on thin histological sections. LDH/EthD-1 is one of the most used dual staining for this purpose. Another advantage is the fact that cryomedium embedded tissue does not have to be immediately stained and imaged. Fluorophores, however, are prone to diffusion over time. This raises the question of how long stained slides remain suitable for imaging. Here, we show that EthD-1 fluorescence remains clearly detectable even 30 days after staining. This extended stability allows researchers to image slides later, reducing the need for immediate analysis and enabling more flexible scheduling.

The second histological method evaluated in this study was MTT combined with DAPI staining. In this protocol, staining is performed prior to OCT embedding. This may cause difficulties in the reuse of leftover cryosections for additional histological analyses such as structural stainings. This method also includes a fluorescent stain (DAPI to stain all nuclei), which we found to remain clearly detectable for at least 30 days post-staining. One limitation of both histology-based assays is that they involve multiple steps, including OCT embedding, freezing, sectioning, staining, and washing. To minimize slow freezing and avoid ice crystal formation, OCT embedded tissue was snap frozen. Nonetheless, each of these steps, despite being standardized, carries the potential to introduce signal distortions or artefacts.

Alongside technical factors, the time required for each technique is an important consideration. Table 2 summarizes the empirical values for how much active time a trained researcher typically spends on sample preparation (e.g., cutting) and staining. Whereas Calcein AM/EthD-1 is time efficient, all steps (including the imaging) must be carried out on the day of harvesting. Histological sections provide a useful alternative, as once embedded, samples can be processed later.

**TABLE 2:** Comparison of methods for cell viability assessment of bovine IVD tissue.

Key Considerations	Calcein AM/EthD-1	LDH/EthD-1	MTT/DAPI
Advantages	Well-established; commercially available	Regional differences easily detectable; resulting cryosections can also be used for further analysis/repetitions; can be reimaged up to 30 days post staining	Regional differences easily detectable; can be reimaged up to 30 days post staining
Drawbacks	Needs immediate processing and imaging; no repetitions possible with same tissue; regional differences hard to assess	Not suited for short term experiments: LDH detectable until 36 h post cell death; increased variability leading to increased sample size needed	Toxic MTT requires careful handling
Recommendation when to use	Short term experiments	Longterm experiments (>5 days)	Short-term experiments where immediate harvesting is not feasible
Active time used (harvesting and staining)	∼1.5 h	∼4 h	∼2.5 h
Suitable positive control	Freshly harvested or Overnight free swelling	Freshly harvested or Overnight free swelling (potentially problematic)	Freshly harvested or Overnight free swelling
Suitable negative control	Negative control 2–4 (harsh treatment needed)	Negative control 2–4 (harsh treatment needed)	Negative control 1 (simple freezing is sufficient)
Additional remarks	ColP pretreatment improves reliability; confocal microscope needed	Positive control pivotal to check staining success; two-channel (fluorescence and BF) microscope needed	Two-channel (fluorescence and BF) microscope needed

In this study, cell viability was assessed by manual counting. To ensure the reliability of this approach, an inter-observer reliability analysis was performed. The analysis demonstrated high inter-rater agreement between observers, confirming that manual counting provided reproducible results for this study.

### 4.2 Positive and negative controls for all techniques

The second aim of this study was to validate positive and negative controls for each technique. Positive controls are used as a benchmark for complete tissue viability. Tissue harvested directly from a healthy, living organism is generally assumed to be viable and therefore suitable as positive control. Importantly, we demonstrated here that not all positive controls are equally suitable for every type of viability assessment.

A note should be made that for the ColP–Calcein AM/EthD-1 staining, the Overnight free swelling group showed ∼10% lower iAF and NP viability compared to the directly harvested group. Though not statistically significant, it may indicate early culture-related effects and should be considered when planning controls.

LDH/EthD-1 staining occasionally suggested non-viability in Overnight free swelling samples, particularly in the oAF. In contrast, ColP-Calcein AM/EthD-1 and MTT/DAPI staining of similarly treated IVDs showed no such discrepancies. This indicates that the apparent cell death is likely due to a technical issue rather than true loss of viability. As only certain IVDs during the same staining exhibited this effect, we hypothesize that the staining is unlikely to be the source. It is more likely to originate during OCT embedding, snap-freezing or sectioning, though the underlying cause remains uncertain. Factors like temperature, humidity, or drying out may play a role, and it should be considered as a weakness of the LDH/EthD-1 method.

Negative controls in viability assays are intended to represent completely non-viable tissue, ensuring the specificity of viability stains. However, such controls are often missing in *ex vivo* IVD studies, and standardized protocols for inducing complete tissue death are lacking. For MTT/DAPI, two simple freeze–thaw cycles (−20 ° ⇔ room temperature, Negative control 1) were sufficient to eliminate MTT staining completely, indicating a loss of mitochondrial activity and thus viability. In contrast, this method was insufficient for Calcein AM/EthD-1, and particularly LDH/EthD-1, where cells continued to exhibit positive staining. This likely reflects the different mechanisms of the stains: MTT is highly sensitive to changes in mitochondrial metabolism, while Calcein AM depends on intracellular esterase activity. Although we observed reduced Calcein AM staining, residual signal remained, especially in the oAF. Mechanistically this suggests partial preservation of esterase activity and membrane integrity even after freeze–thawing. Concerning LDH, previous studies have noted that LDH activity can persist for up to 36 h after cell death, making it a slower-reacting viability indicator compared to MTT or Calcein AM (Gantenbein-Ritter et al., 2008; Stoddart et al., 2006). We here showed a similar trend, as tissue from Negative control 1 group stained positively for LDH. For short-term experiments, MTT/DAPI and Calcein AM/EthD-1 are therefore preferable. Previously, very harsh treatment (2x freeze-thaw (−193° ⇔ 37 °C) and/or heat incubation (56 °C)) have been shown to be necessary to effectively obtain dead cells in human cancellous bone tissue (Stoddart et al., 2006; Stoddart, 2011). Interestingly, our results confirm that remaining LDH positivity after cell death is not solely time-dependent but also influenced by the method of cell killing. When tissues were snap-frozen and subsequently heat-incubated in liquid, LDH activity was already completely abolished within 24 h (Negative control 2). We propose that harsh treatment (snap-freezing and thawing) led to immediate necrosis, with more extensive membrane disruption. Combined with liquid immersion and heat treatment, LDH was efficiently released or inactivated, producing a loss of detectable staining within 24 h.

Taken together, both positive and negative controls are essential for properly setting imaging parameters such as laser intensity, exposure time, and gain. Relying solely on positive controls or even experimental groups to adjust these settings can lead to underexposure of cell death markers (Supplementary Figure S5). By using both positive and negative controls to define imaging settings, bias of overestimating tissue viability can be lessened.

### 4.3 Choosing the right viability assessment

**FIGURE 5:**
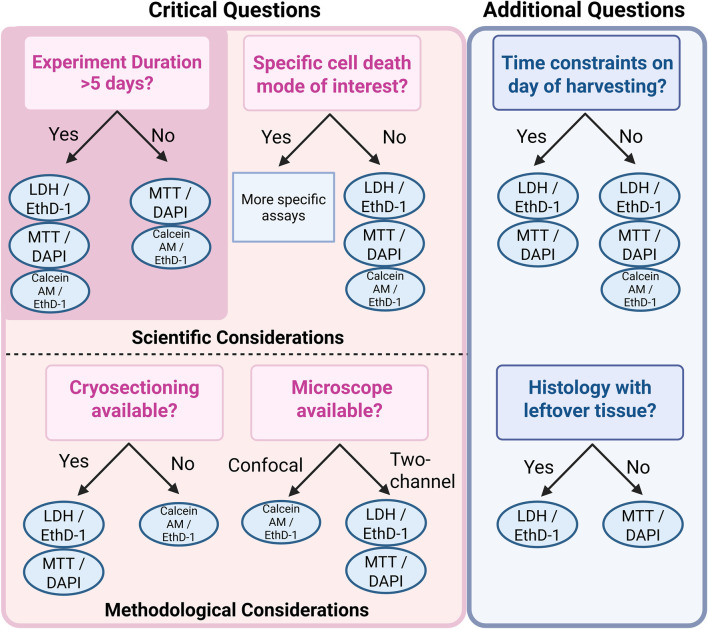
Guiding questions to support informed decision-making when selecting a cell viability assessment method for IVD tissue.

Overall, each viability assay has its own advantages and drawbacks, and an appropriate assay should be chosen based on the experimental set-up. Based on existing knowledge from literature and our results, we compiled the key considerations for selecting an appropriate assay in Table 2 and Figure 5.

A notable limitation of the LDH/EthD-1 method is that LDH can remain detectable in cells for up to 36 h post–cell death. To compensate for this, incubating IVDs for an additional 1–3 days after the experimental endpoint could be a potential correction strategy. However, the exact duration required for effective correction remains unclear and might also depend on the main mode of cell death. Furthermore, if discs are intended for analyses beyond viability (e.g., gene expression), additional incubation could alter or mask experimental outcomes. By restricting LDH/EthD-1 use to longer-term experiments (>5-day organ cultures), these issues can be largely avoided.

In addition to the three core viability assessment techniques discussed here, other methods specifically targeting apoptotic cells are available. Examples include caspase staining (e.g., cleaved caspase-3), TUNEL assay (Terminal deoxynucleotidyl transferase dUTP Nick End Labelling), and Annexin V staining, all of which have been applied to assess cell viability in cartilage or IVD tissue samples (Thomas et al., 2007; Emans, Bulstra, and Kuijer, 2005; Wangler et al., 2019; Su et al., 2021). When apoptosis is the primary focus, these assays provide more specific answers compared to the more traditional viability assays presented here.

This study focuses on the use of bovine IVD tissue, which represents a highly specific model system. When considering the translation of our results to human IVD tissue, several points must be noted. Firstly, all stains are restricted to freshly obtained tissue and are not suitable for application to previously frozen tissue. Both, MTT/DAPI and Calcein AM/EthD-1 have been successfully used to assess cell viability in human organ culture models (Walter et al., 2014; Rosenzweig et al., 2016). Rosenzweig et al. report only NP and iAF viability using Calcein AM/EthD-1. Therefore, it remains unclear whether similar staining penetration issues occurred in oAF as observed in our study. LDH/EthD-1 offers the key advantage that tissue can be cryopreserved upon receipt and stained later, whereas Calcein AM/EthD-1 and MTT/DAPI require immediate incubation. This would make LDH/EthD-1 an interesting candidate for use in human IVD tissue as well. However, the differences in matrix composition between bovine and human IVDs may require specific optimization of stain concentration and incubation times. Nonetheless, we expect our findings and practical recommendations to be translatable to human tissue. Furthermore, these results may extend to other systems, such as 3D cell-laden scaffolds and dense, collagen-rich soft tissues like tendons.

### 4.4 Limitations

This study has several limitations. Although bovine tails are widely used in IVD research, they only represent a specific tissue type. While our findings may translate to human IVD tissue or other dense soft tissues, this was not specifically evaluated here. Sample sizes in some groups were small (as low as n = 3). In addition, all experiments were conducted at a single institute, and differences in handling at other sites may affect reproducibility. A further limitation is the lack of explanation for the large standard deviation observed for LDH/EthD-1 staining in the Overnight free swelling oAF group; this should be investigated in future studies.

### 4.5 Conclusion

In this study, we present detailed protocols for using Calcein AM/EthD-1, LDH/EthD-1, and MTT/DAPI to assess cell viability in bovine IVD tissue. The methods are compared and discussed based on our results and existing literature. We hope that this work will support researchers in selecting the most suitable viability assay for their specific research questions and experimental setups.

## Data Availability

The original contributions presented in the study are included in the article/Supplementary Material, further inquiries can be directed to the corresponding author.
