# Supplementary material for: Optimization and comparison of different methods for assessing cell viability in intervertebral disc organ cultures
Source: Front Bioeng Biotechnol. 2026 Apr 29;14:1796998. doi: 10.3389/fbioe.2026.1796998 (PMC13167986; doi:10.3389/fbioe.2026.1796998)

Supplementary Material

# Supplementary Figures

#
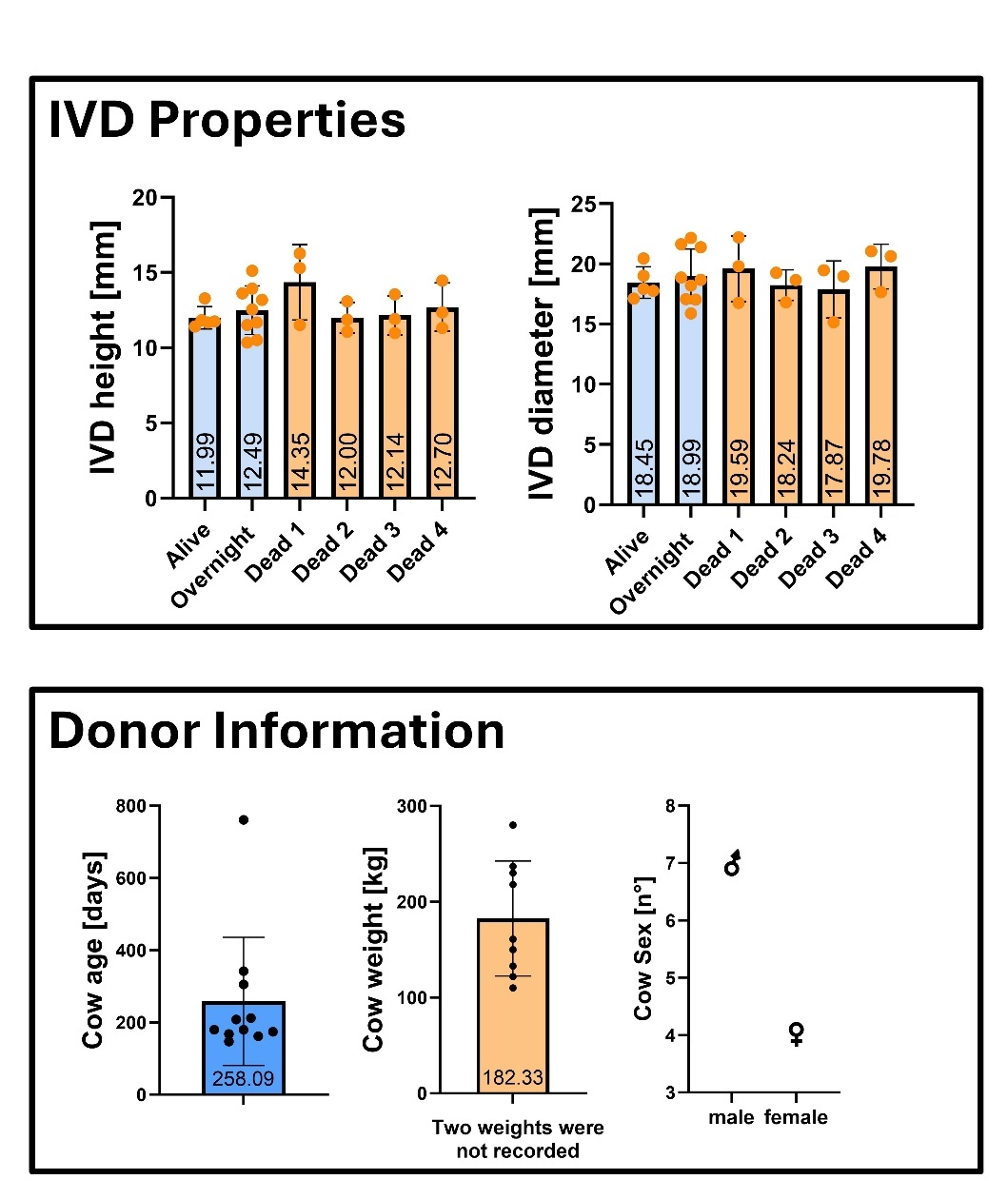
Supplementary Figure 1. IVD properties and donor information of bovine tails used.


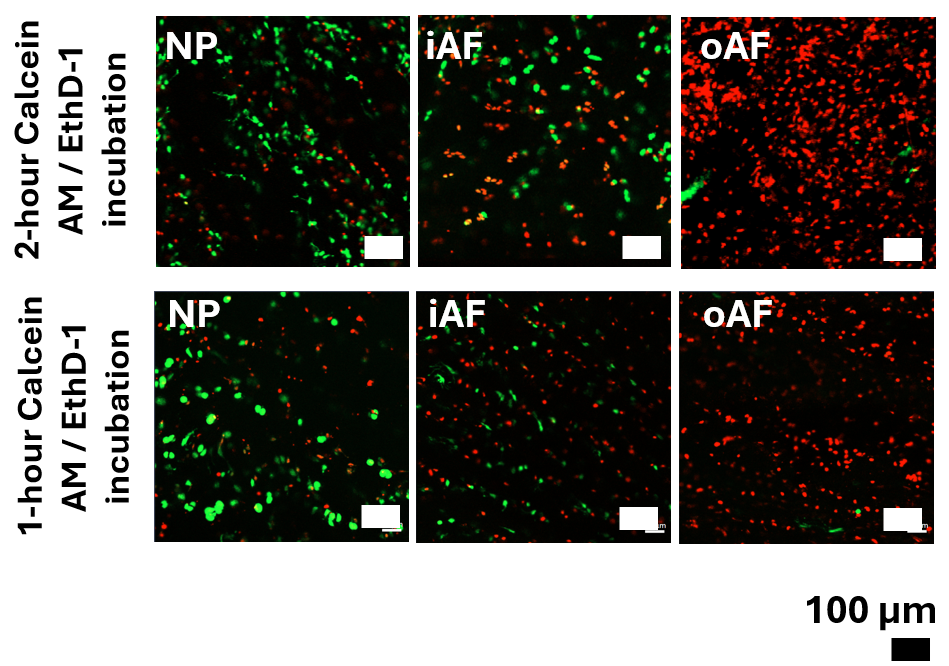
**Supplementary Figure 2.** Comparison of 2-hour vs. 1-hour incubation. This comparison was made with Overnight free swelling samples not undergoing ColP pre-treatment. Therefore, the oAF appears completely dead. No difference in staining was observed between a 2-hour and 1-hour incubation qualitatively, and with the interest in keeping staining time short, all following experiments were conducted using 1-hour incubations. Green = Calcein AM positive; Red = EthD-1 positive.

**Supplementary Figure 3.** Stability of DAPI fluorescence strength over 30 days. No clear decline in the DAPI signal was observed over the course of 30 days. This is shown here for NP tissue, but similar results were obtained for iAF and oAF samples. Dark blue = MTT positive, light blue = DAPI positive.


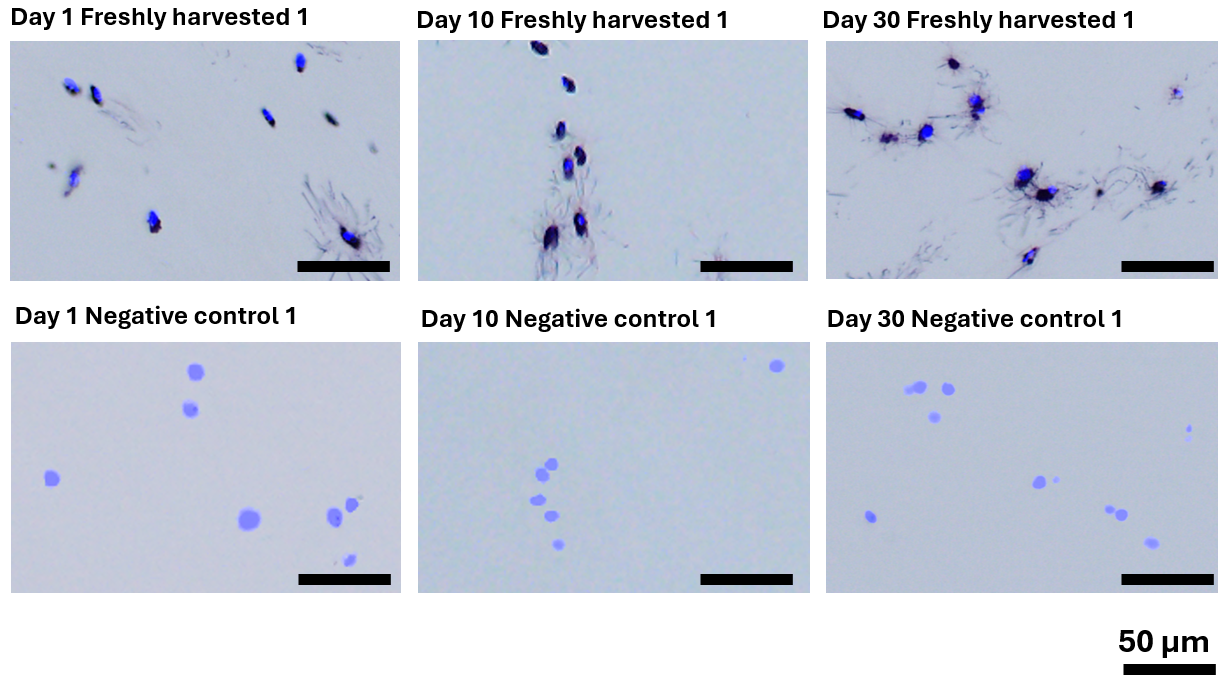


**Supplementary Figure 4.** Observations for Overnight free swelling IVDs. Representative images to describe discrepancies in results when staining sections from the same disc with LDH / EthD-1 for the Overnight free swelling group. Of the 9 stained samples, 3 showed this phenomenon. Although the samples were not all harvested and cut simultaneously, they were all stained at the same time and originated from overnight free swelling IVDs. Below are ColP-Calcein AM / EthD-1 images from a “bad” sample for reference, showing high viability. For LDH / EthD-1: Blue = LDH positive, Orange = EthD-1 positive. For Calcein AM / EthD-1: Green = Calcein AM positive; Red = EthD-1 positive.


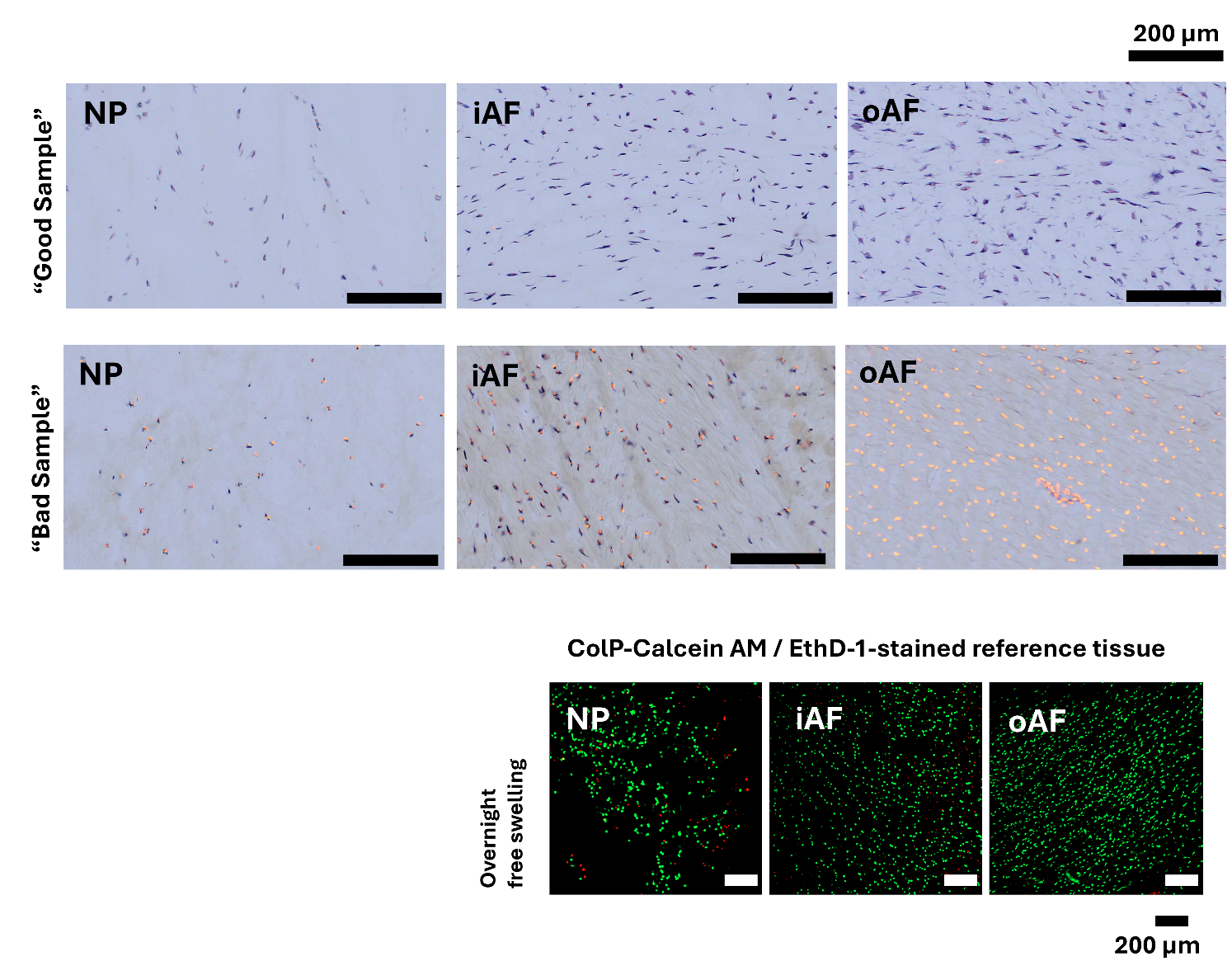


**Supplementary Figure 5.** Representative images from the Freshly harvested and Negative control 2 groups illustrate that samples can still be imaged up to 30 days after staining, provided appropriate exposure settings are used. Although all regions were imaged, only the NP region is shown here. All regions exhibited comparable results. Green, blue, orange and purple arrows are used to facilitate comparison of individual cells across the images. This comparison also highlights the critical role of both positive and negative controls in establishing appropriate imaging parameters. If only positive controls are considered, the EthD-1 exposure is more likely to be set too low. With the adjusted exposure, dead cells become much easier to identify and count (e.g., green arrow in the top row). Blue = LDH positive, Orange = EthD-1 positive.


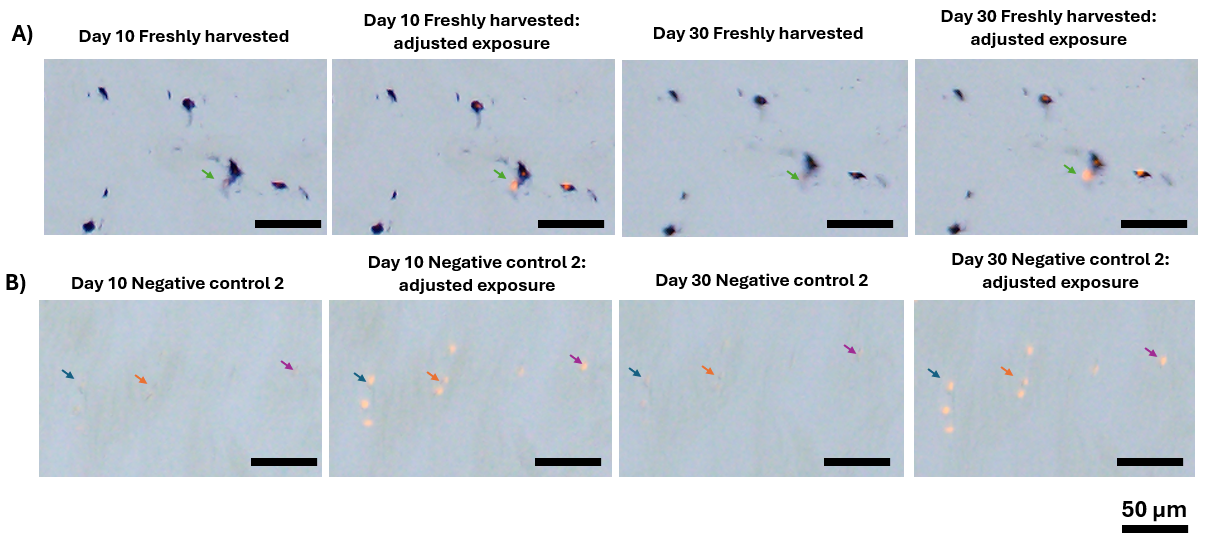


**Supplementary Figure 6.** Uncropped 300 dpi representative images from the Freshly harvested group for all disc regions after ColP-Calcein AM / EthD-1 staining (Scalebar = 100 µm).

NP


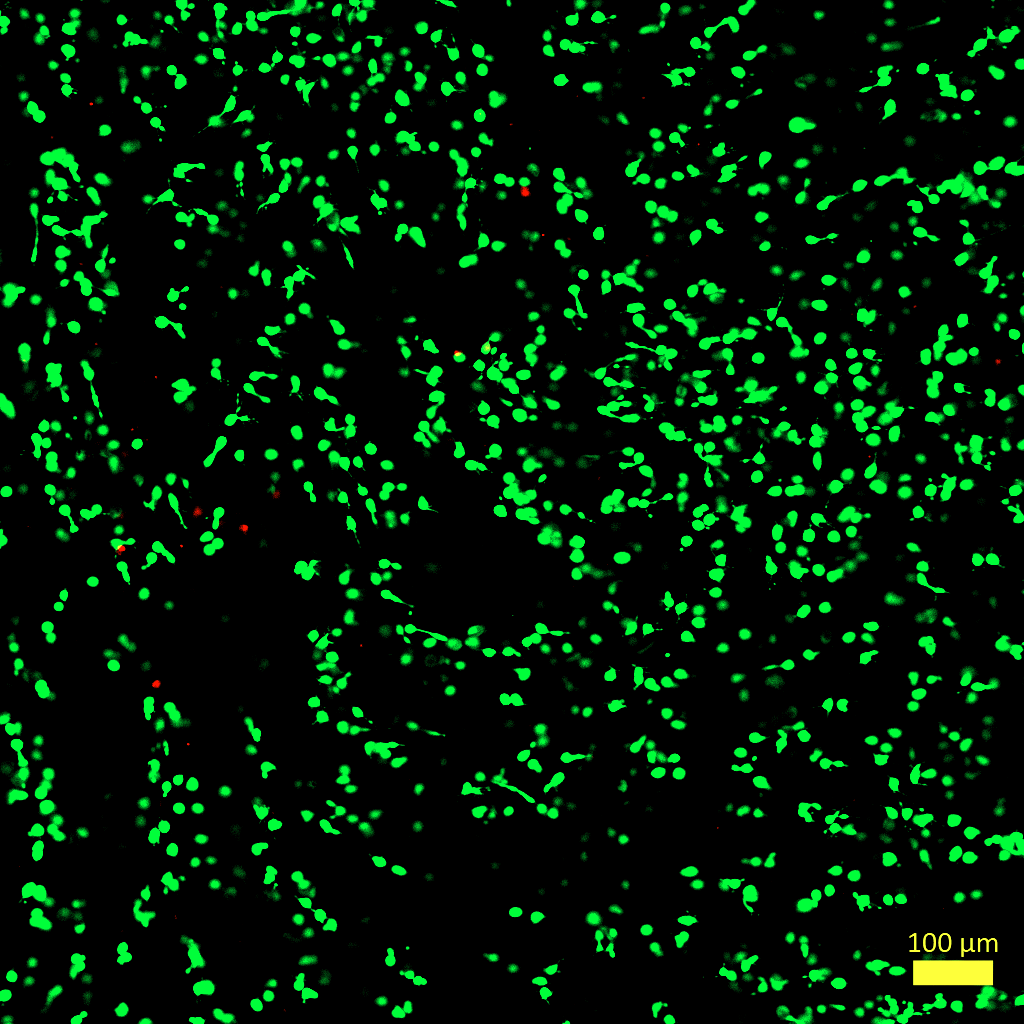


iAF


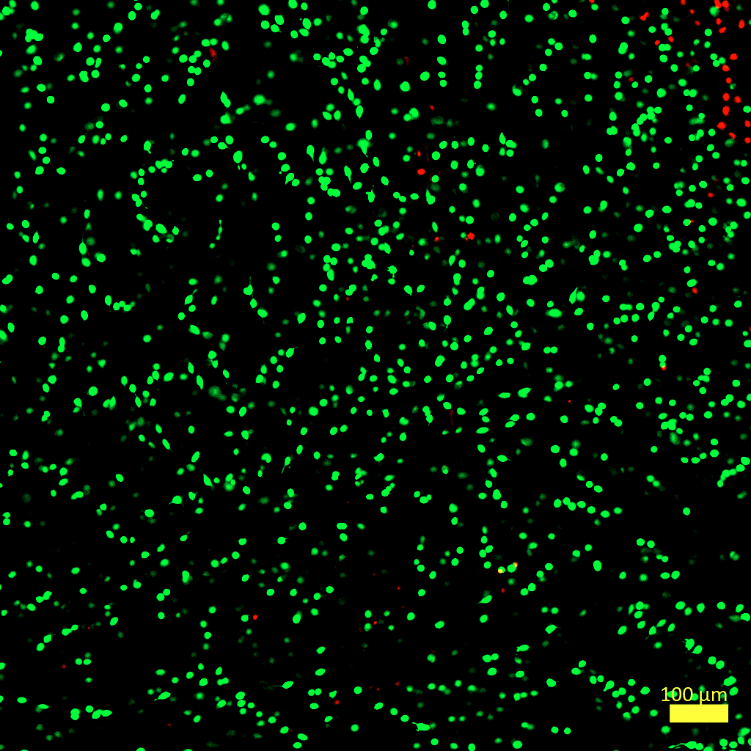


oAF


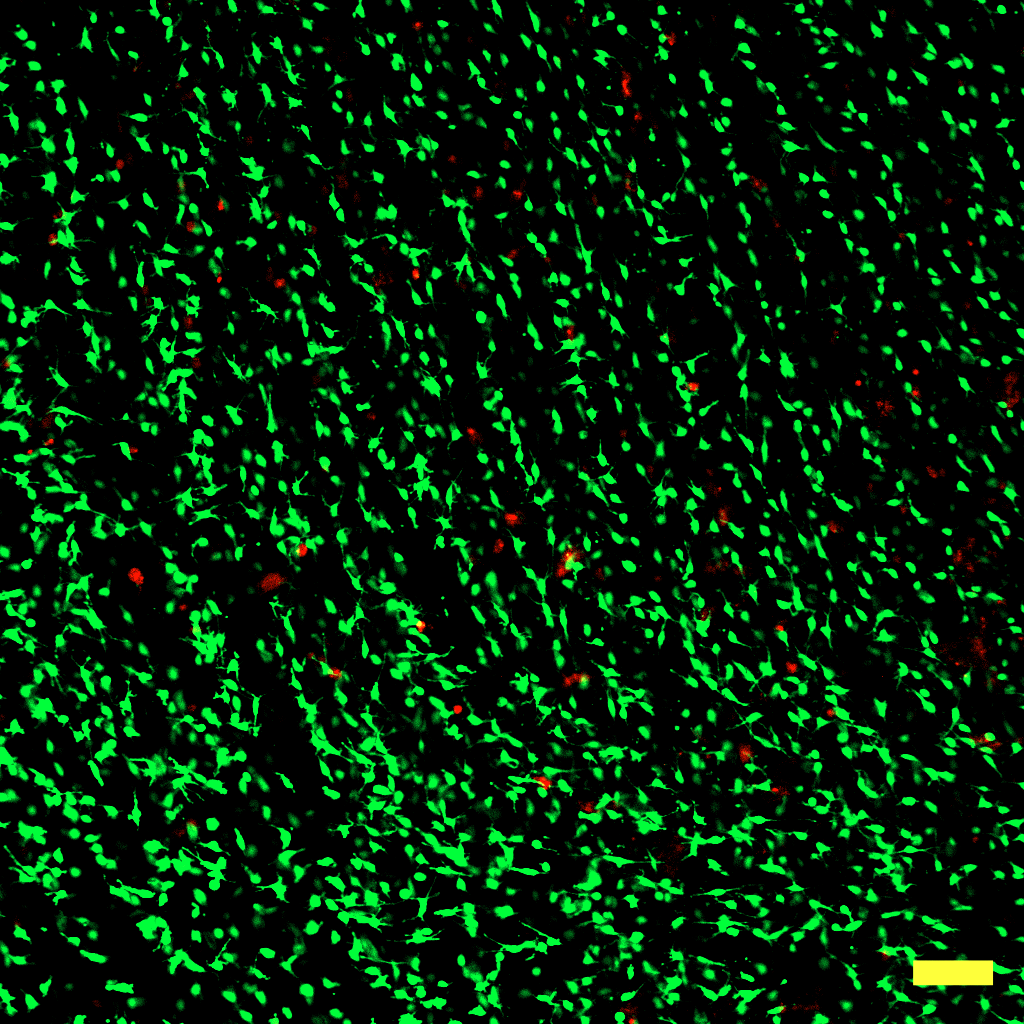


**Supplementary Figure 7.** Uncropped 300 dpi representative images from the Negative control 2 group for all disc regions after ColP-Calcein AM / EthD-1 staining (Scalebar = 100 µm).

NP


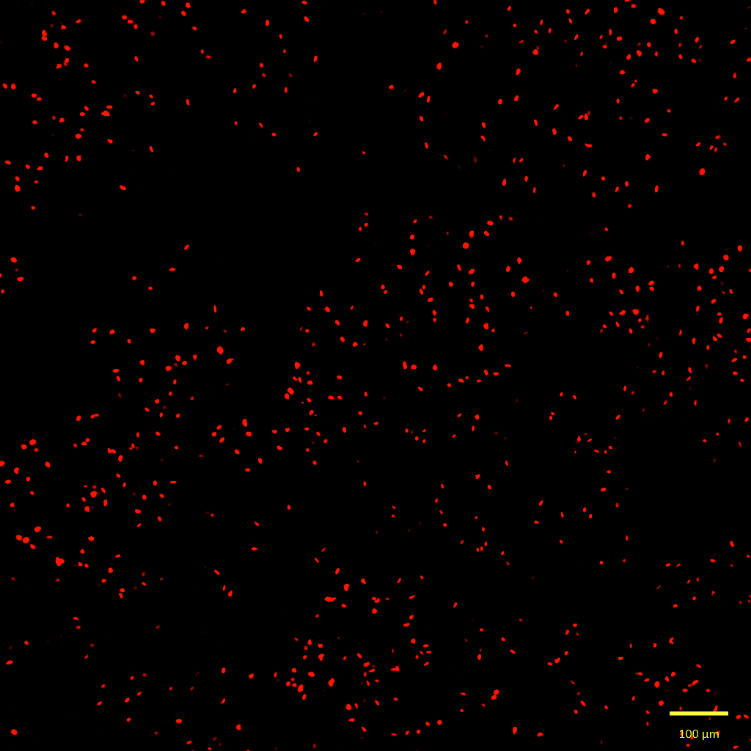


iAF


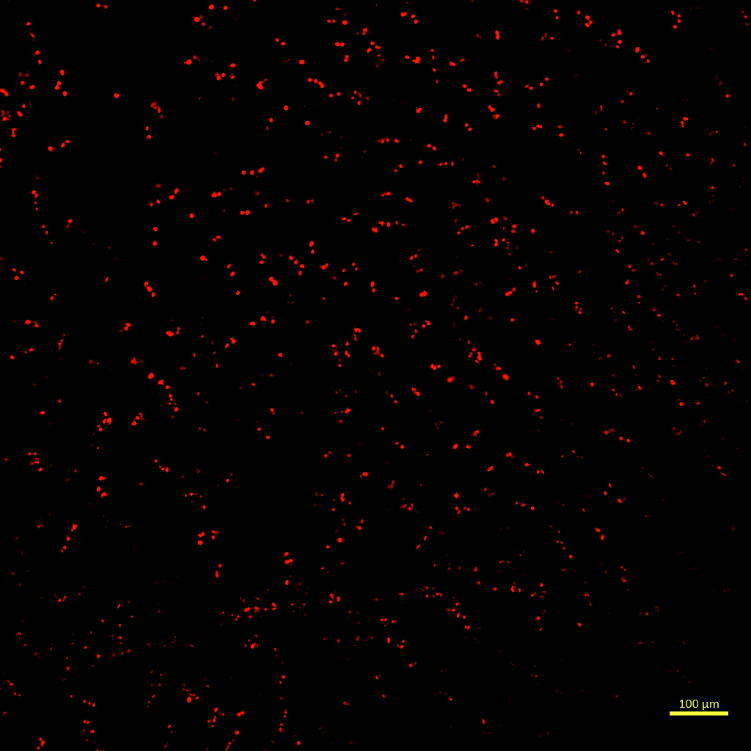


oAF


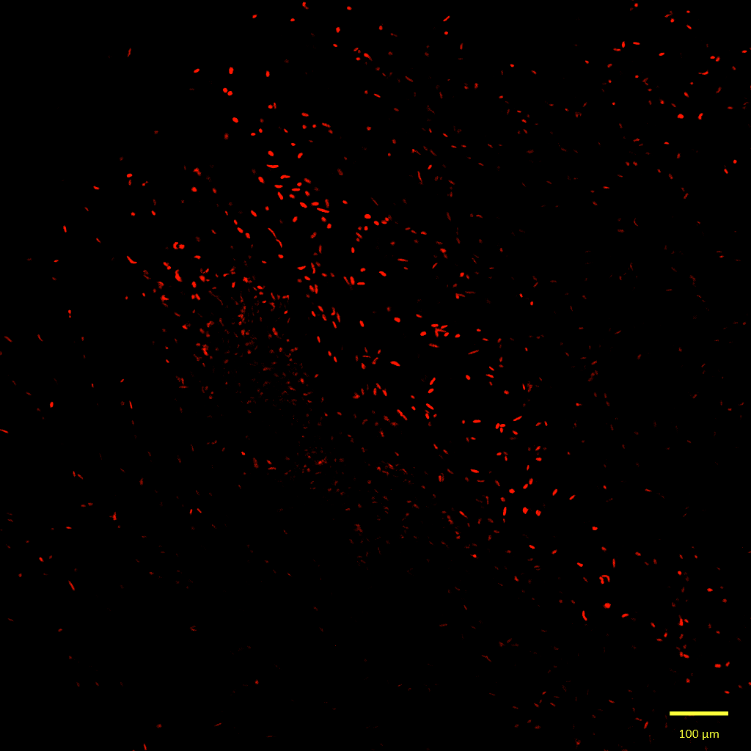


**Supplementary Figure 8.** Uncropped 300 dpi representative images from the Freshly harvested group for all disc regions after MTT / DAPI staining (Scalebar = 200 µm).

NP


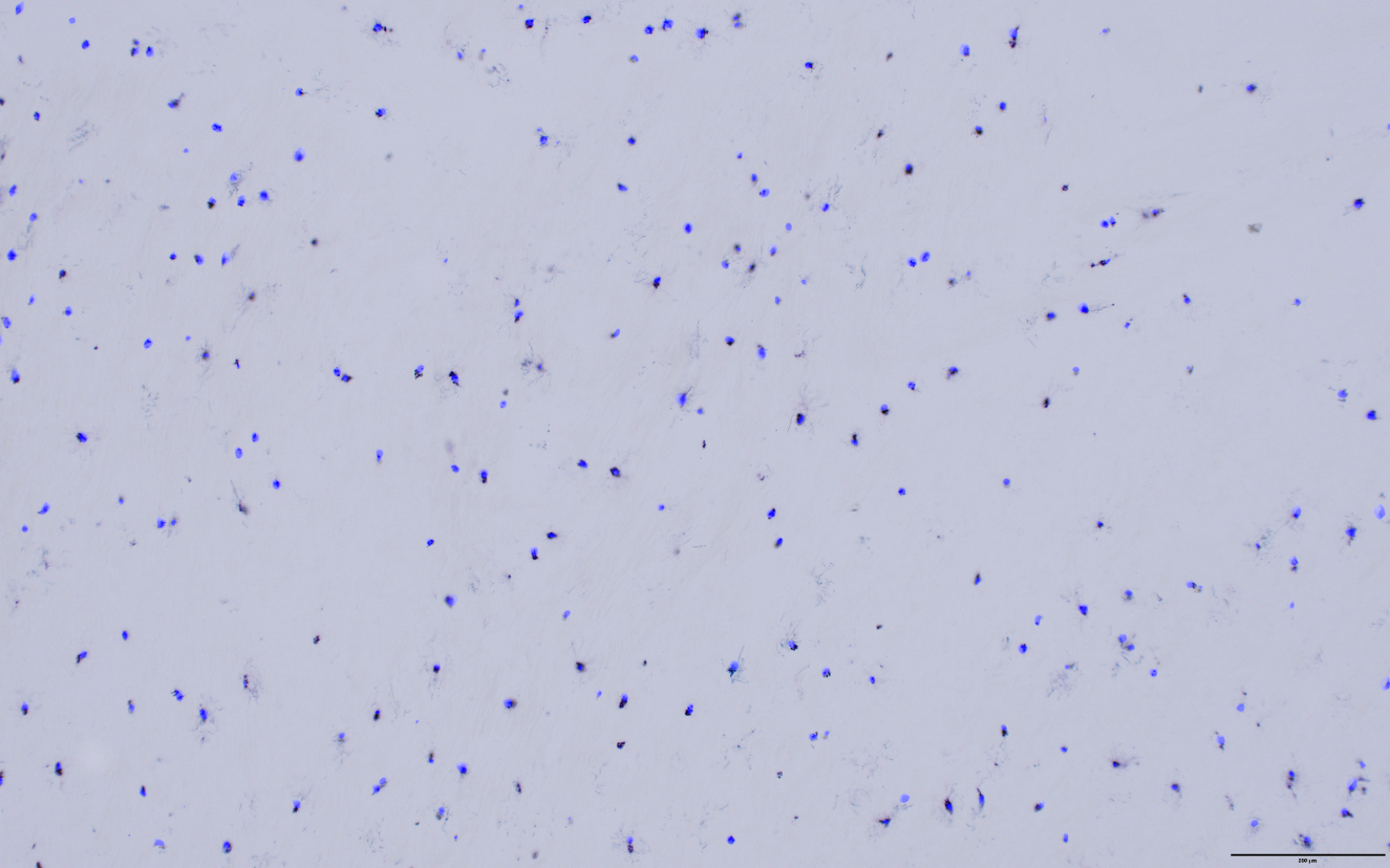


iAF


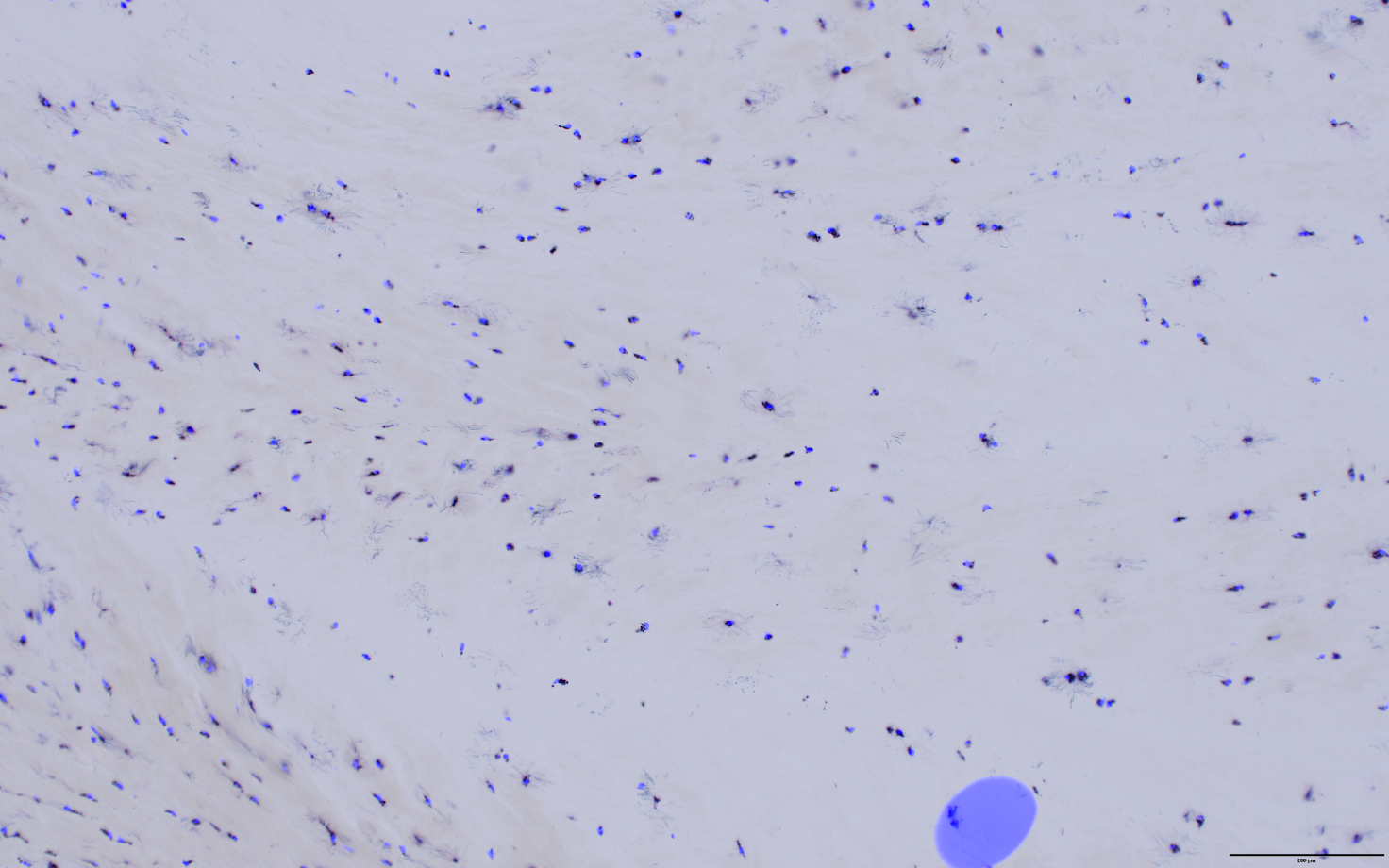


oAF


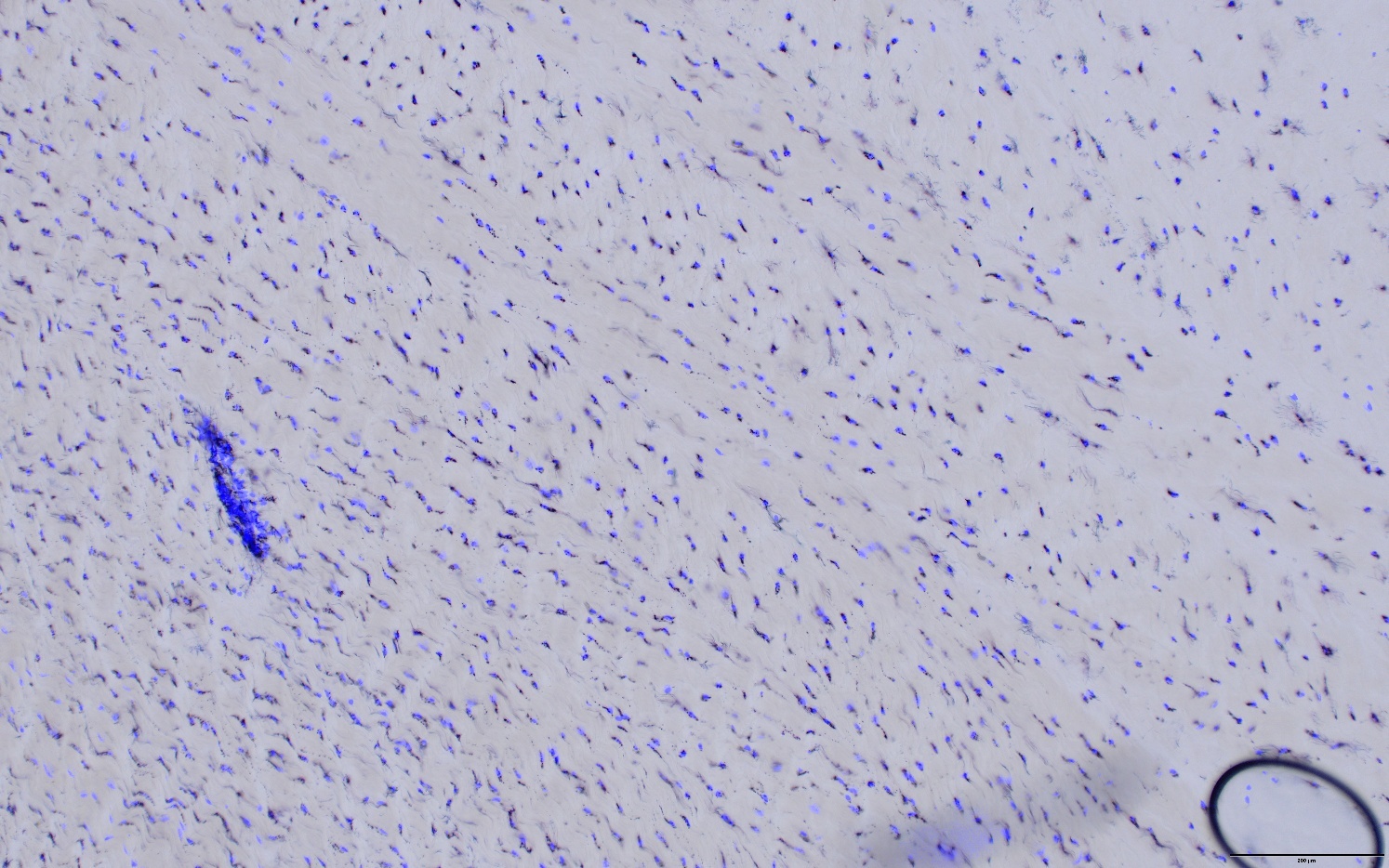


**Supplementary Figure 9.** Uncropped 300 dpi representative images from the Negative control 1 group for all disc regions after MTT / DAPI staining (Scalebar = 200 µm).

NP


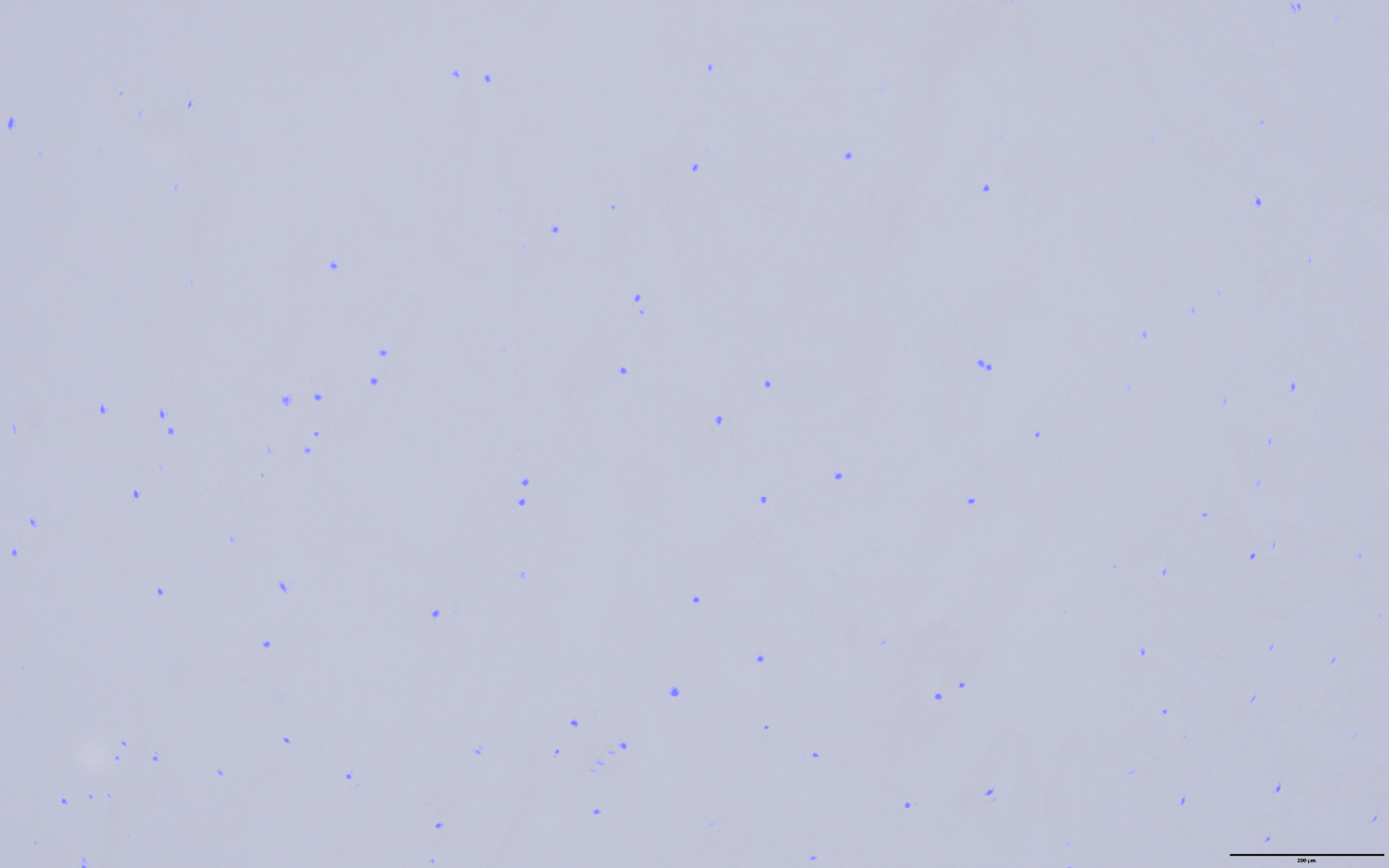


iAF


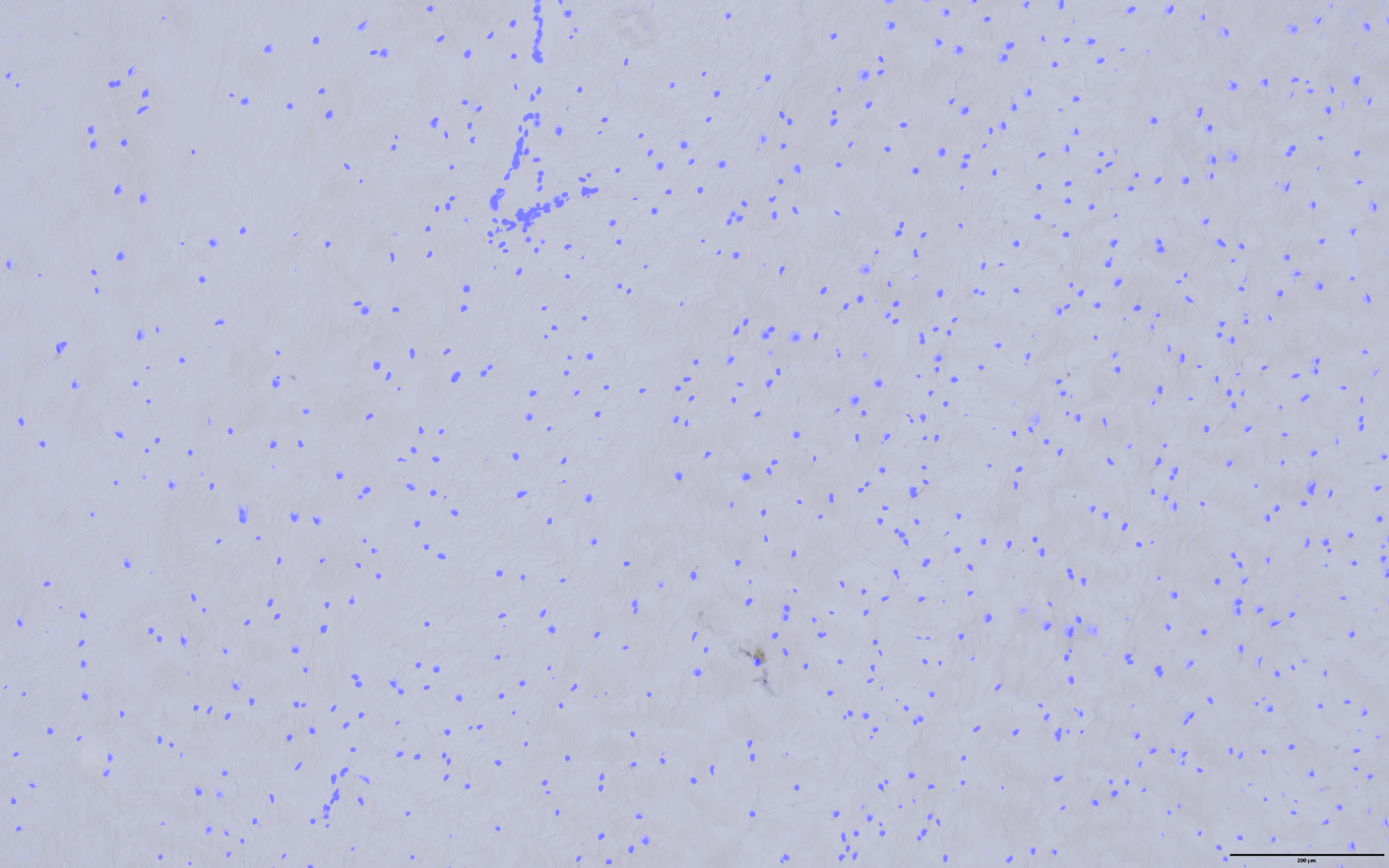


oAF


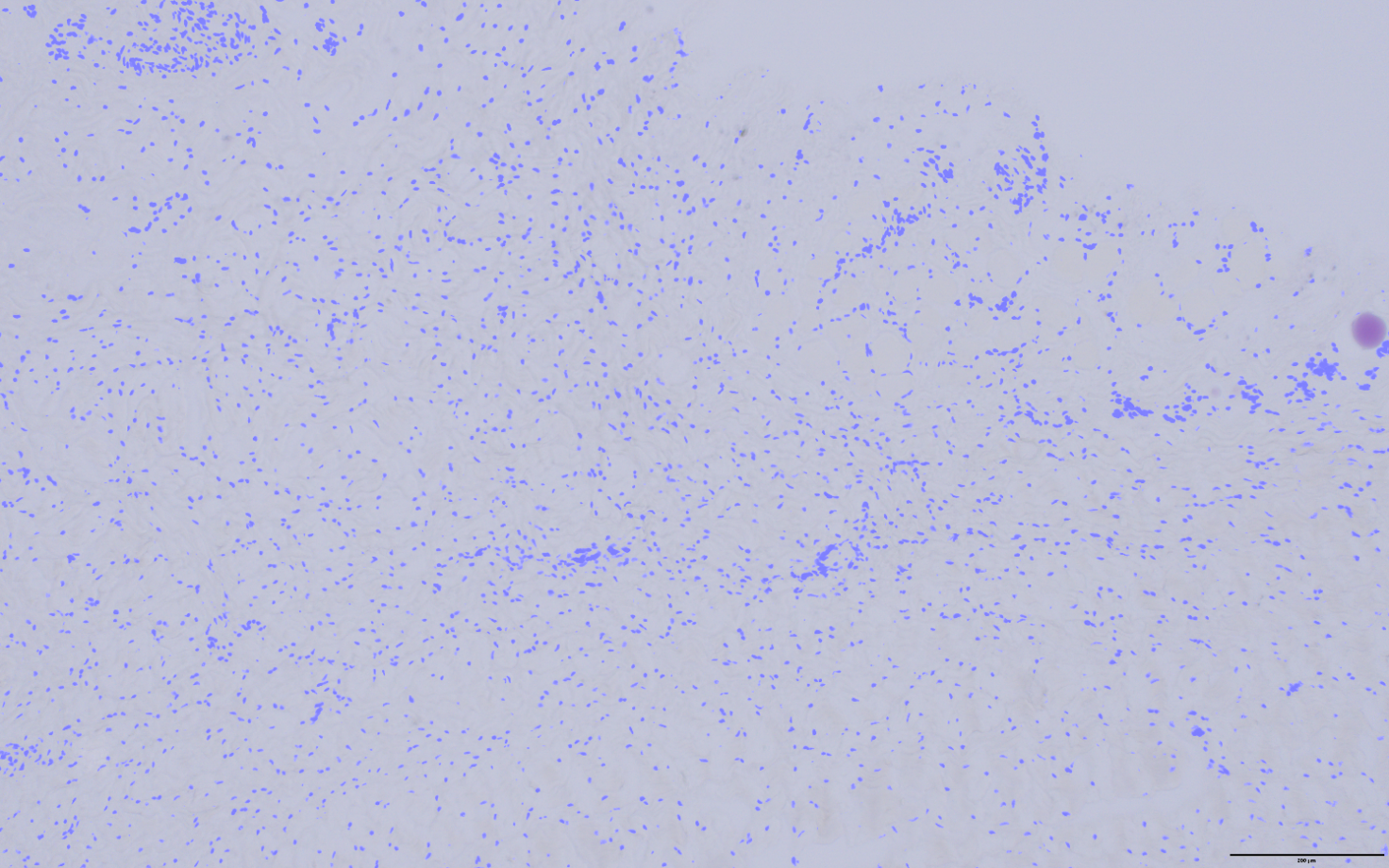


**Supplementary Figure 10.** Uncropped 300 dpi representative images from the Freshly harvested group for all disc regions after LDH/EthD-1 staining (Scalebar = 200 µm).

NP


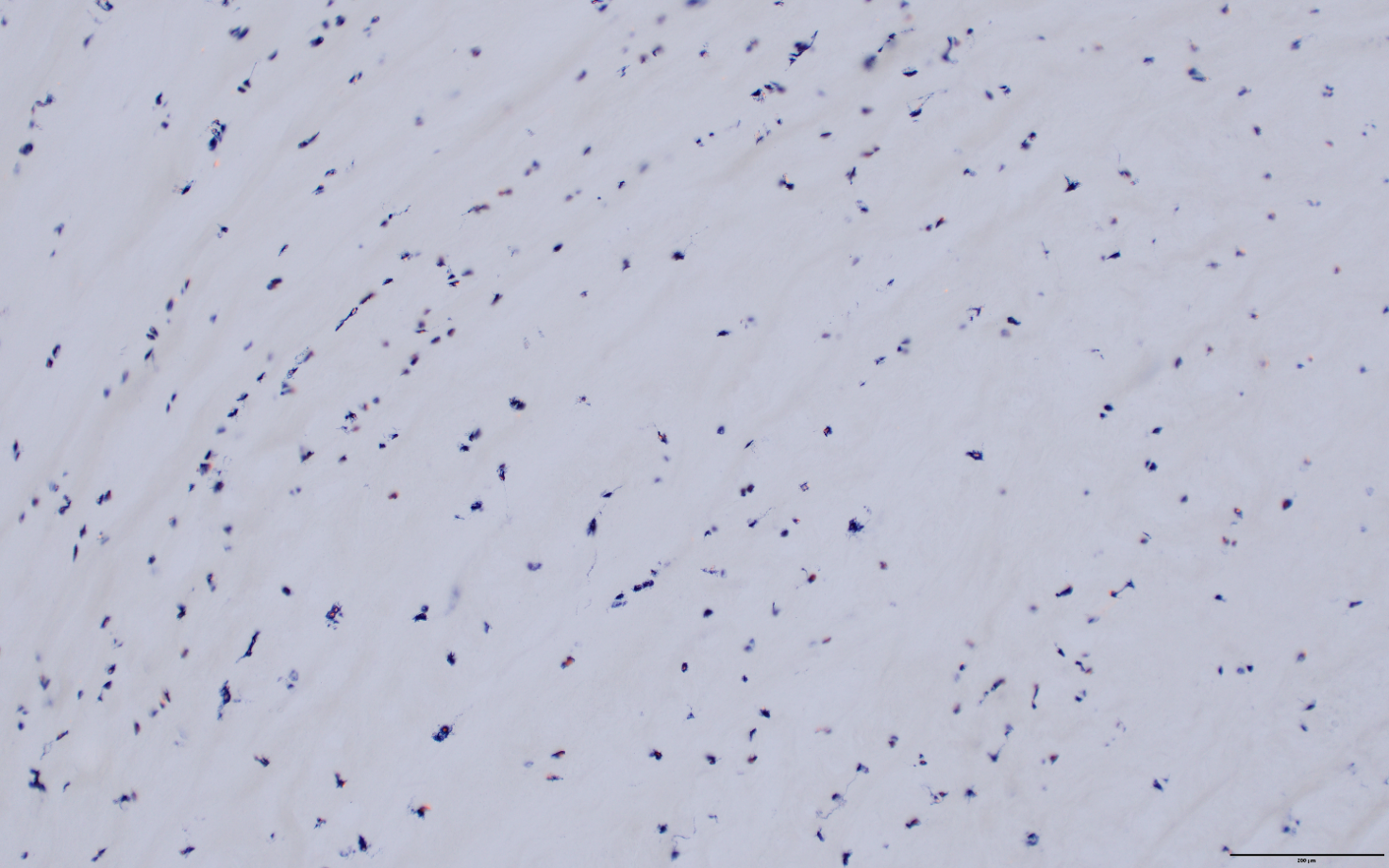


iAF


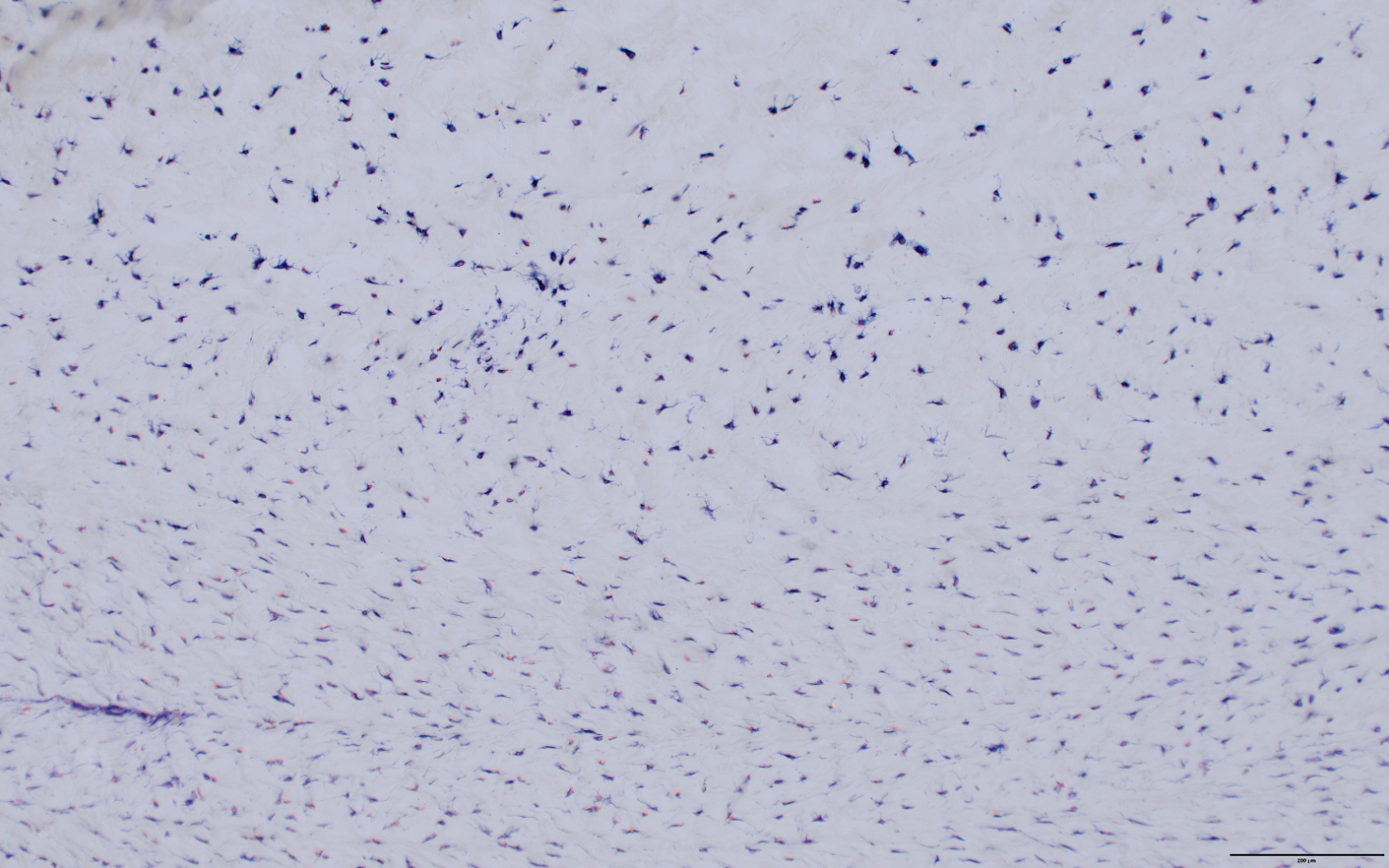


oAF


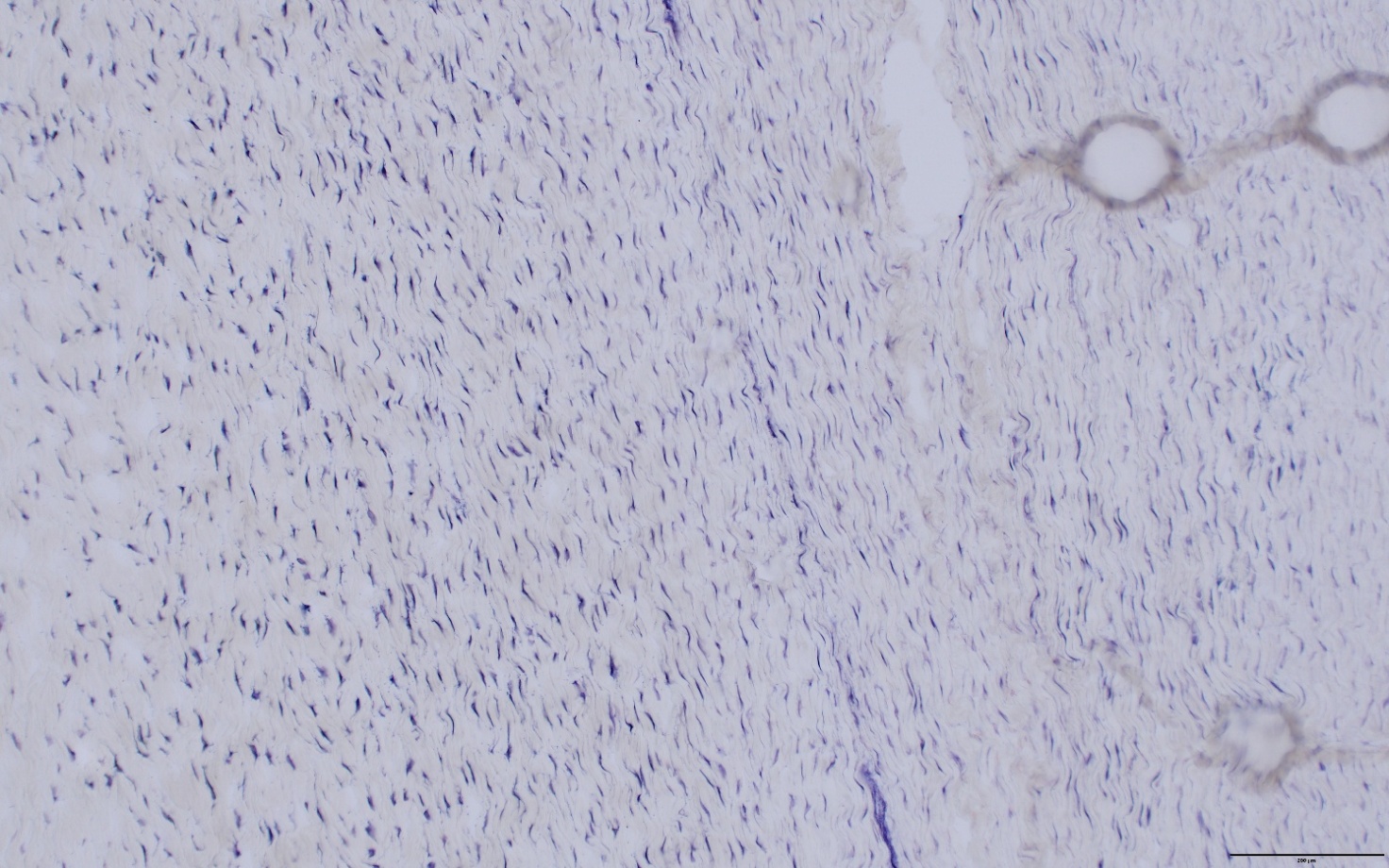


**Supplementary Figure 11.** Uncropped 300 dpi representative images from the Negative control 2 group for all disc regions after LDH/EthD-1 staining (Scalebar = 200 µm).

NP


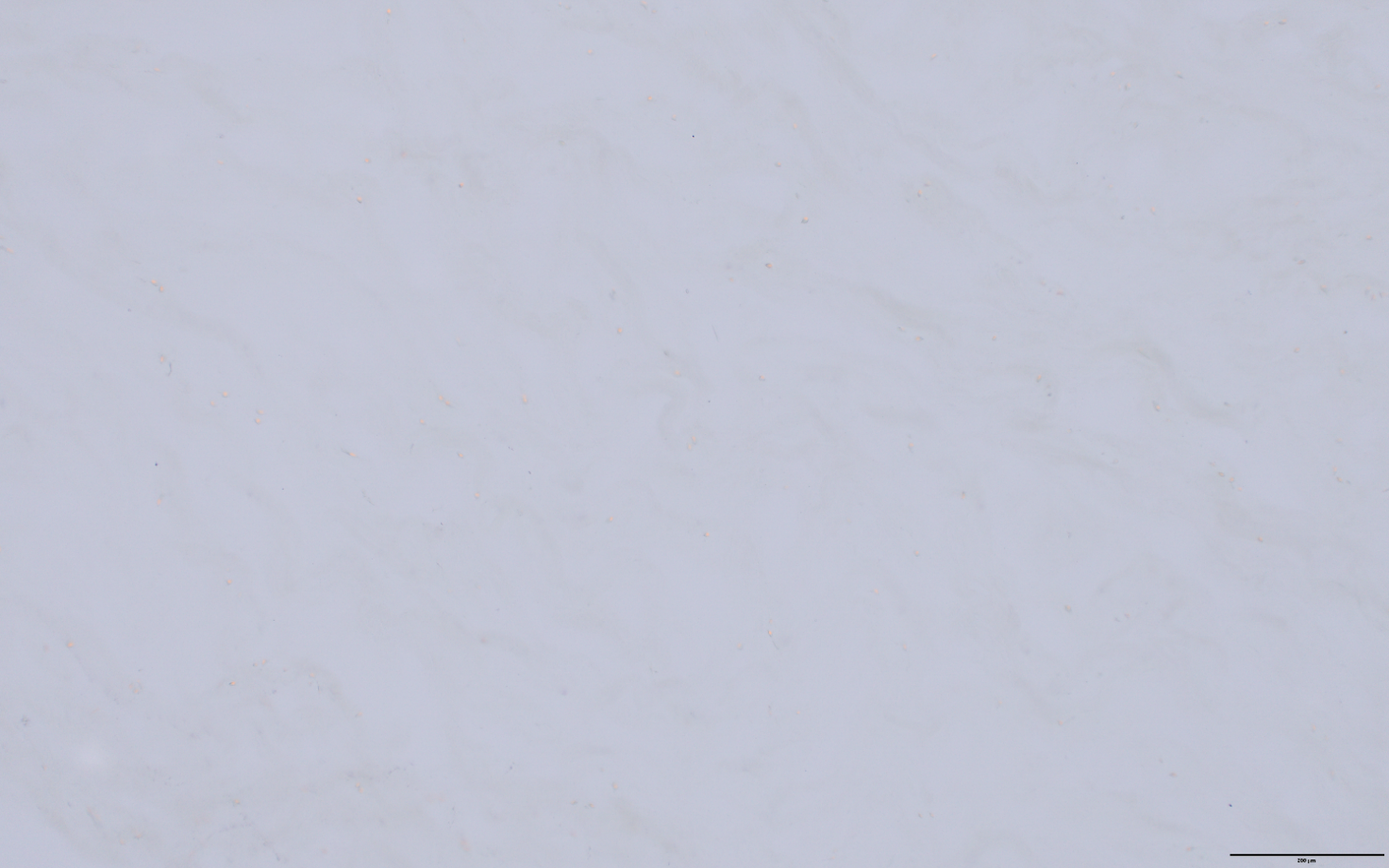


iAF


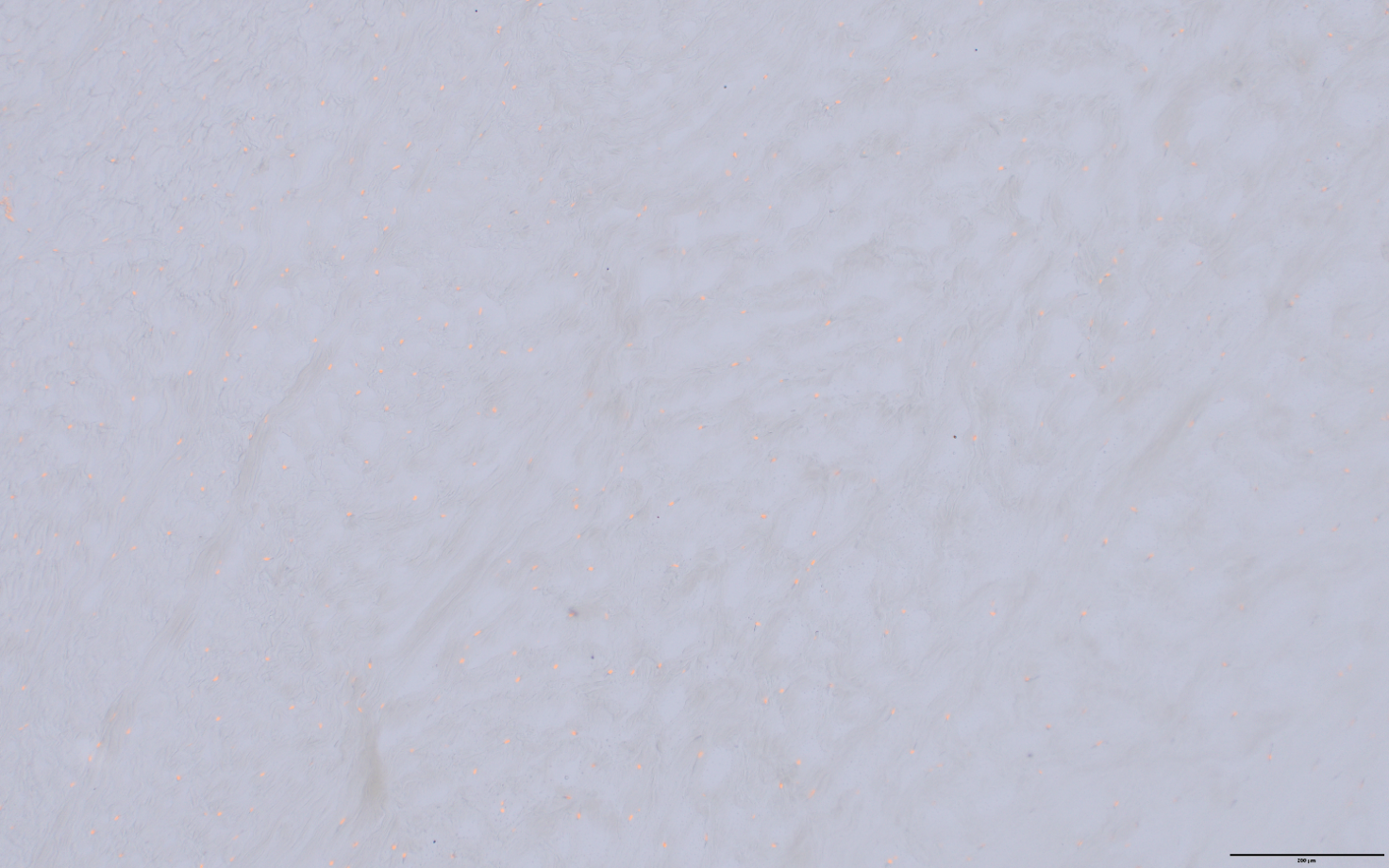


oAF


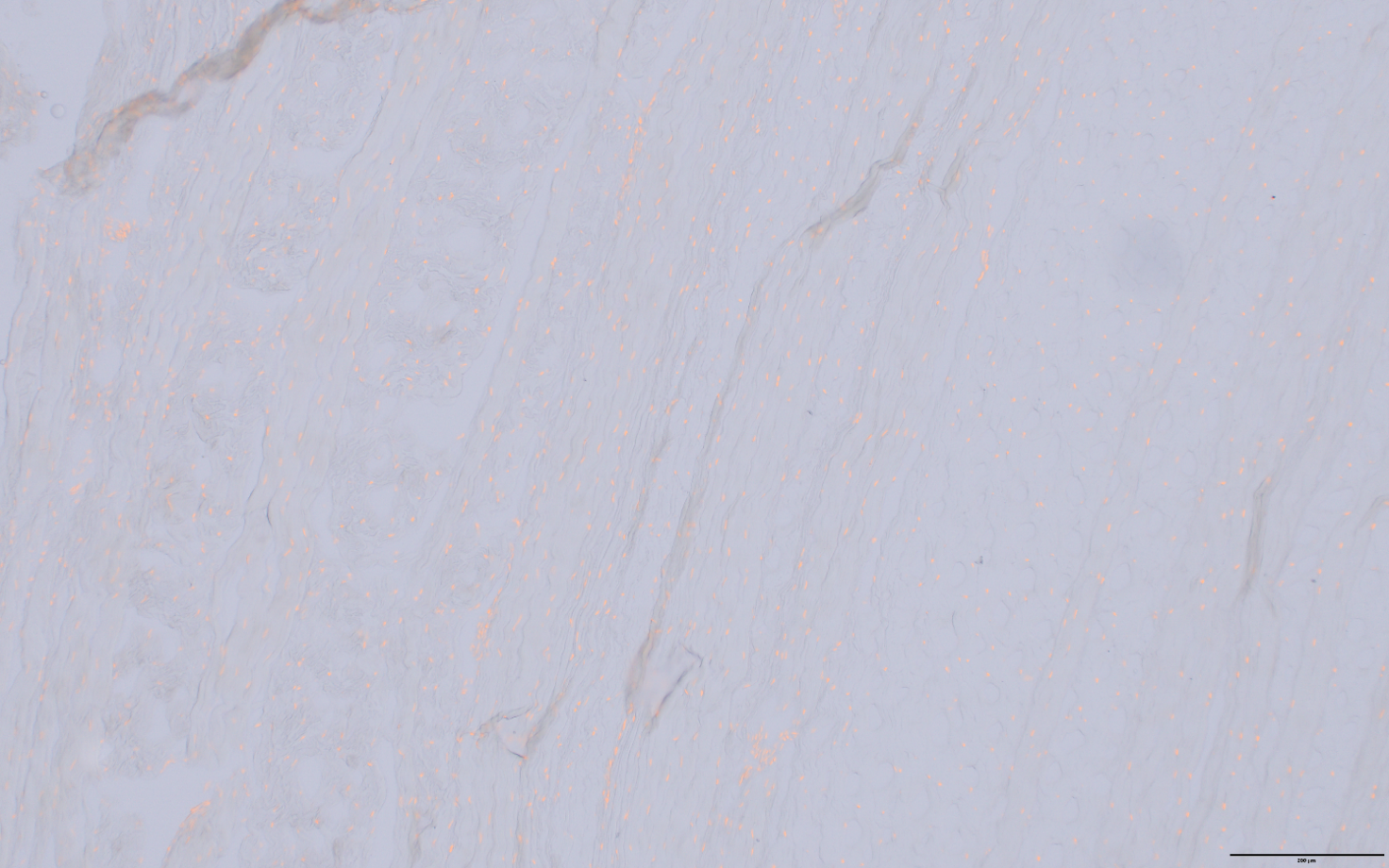


**Supplementary Figure 12.** Bland-Altman plot of inter-observer reliability assessment.


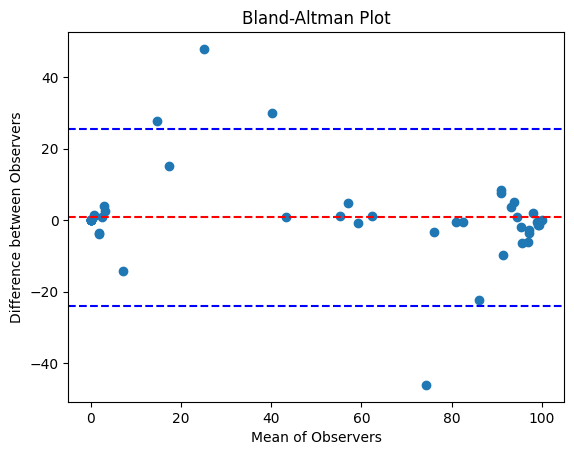

Supplement: Supplementary file 1 [file Supplementaryfile1.docx]
